# CYYR1 promotes the degradation of the E3 ubiquitin ligase WWP1 and is associated with favorable prognosis in breast cancer

**DOI:** 10.1016/j.jbc.2024.107601

**Published:** 2024-07-24

**Authors:** Tiphaine Perron, Mathieu Boissan, Ivan Bièche, Laura Courtois, Florent Dingli, Damarys Loew, Mouna Chouchène, Sabrina Colasse, Laurence Levy, Céline Prunier

**Affiliations:** 1 Sorbonne Université, INSERM UMR_S 938, Centre de Recherche Saint-Antoine, CRSA, Paris, France; 2 APHP, Hôpitaux Universitaires Pitié-Salpêtrière-Charles Foix, Laboratoire de Biochimie Endocrinienne et Oncologique, Oncobiologie Cellulaire et Moléculaire, Paris, France; 3 Department of Genetics, Institut Curie, Université Paris Cité, Paris, France; 4 CurieCoreTech Mass Spectrometry Proteomics, Institut Curie, PSL Research University, Paris, France

**Keywords:** E3 ubiquitin ligase, WWP1, CYYR1, ubiquitination, breast cancer

## Abstract

Ubiquitination plays a crucial role in cellular homeostasis by regulating the degradation, localization, and activity of proteins, ensuring proper cell function and balance. Among E3 ubiquitin ligases, WW domain-containing protein 1 (WWP1) is implicated in cell proliferation, survival, and apoptosis. Notably *WWP1* is frequently amplified in breast cancer and associated with poor prognosis. Here, we identify the protein cysteine and tyrosine-rich protein 1 (CYYR1) that had previously no assigned function, as a regulator of WWP1 activity and stability. We show that CYYR1 binds to the WW domains of the E3 ubiquitin ligase WWP1 through its PPxY motifs. This interaction triggers K63-linked autoubiquitination and subsequent degradation of WWP1. We furthermore demonstrate that CYYR1 localizes to late endosomal vesicles and directs polyubiquitinated WWP1 toward lysosomal degradation through binding to ANKyrin repeat domain-containing protein 13 A (ANKRD13A). Moreover, we found that CYYR1 expression attenuates breast cancer cell growth in anchorage-dependent and independent colony formation assays in a PPxY-dependent manner. Finally, we highlight that *CYYR1* expression is significantly decreased in breast cancer and is associated with beneficial clinical outcome. Taken together our study suggests tumor suppressor properties for CYYR1 through regulation of WWP1 autoubiquitination and lysosomal degradation.

Posttranslational modification of proteins through ubiquitination plays an important role in various essential biological processes (1). Ubiquitination consists of the fixation of one molecule (monoubiquitination) or a chain of ubiquitins (polyubiquitination) on a lysine residue of the targeted protein. Polyubiquitination occurs by multiple rounds of ubiquitination on the seven lysine residues (K6, K11, K27, K29, K33, K48, and K63), or the N-terminal methionine (M1) residue of ubiquitin. Ubiquitination creates additional interacting surfaces that lead to various outcomes for the targeted protein depending of the linkage. The most dramatic outcome of ubiquitination is to direct the targeted protein toward degradation, either through the proteosome *via* K48- or K11-linked polyubiquitination, or through the endolysosomal compartment *via* K63-linked polyubiquitination (2). Ubiquitination is a highly regulated process that involves the coordinated activity of a ubiquitin activating E1, a conjugating E2, and a ligating E3 enzymes. Among these enzymes, E3s are crucial in ensuring the specificity of the ubiquitination process, as they are responsible for recognizing specific substrate proteins and facilitating the transfer of ubiquitin molecules to precise lysine residues on those substrates. There are more than 600 E3 ligases in humans that are divided in three main types according to their ubiquitination domain: the homologous to E6AP C terminus (HECT) domain, the really interesting new gene (RING) domain, and the RING-Between-RING (RBR) domain E3 ligases (3). With 28 members, the HECT E3 ubiquitin ligases constitutes a small family of highly conserved enzymes involved in a wide range of pathophysiological processes (4). Among these HECT enzymes, the neural precursor cell-expressed developmentally downregulated 4 (NEDD4) subfamily is the best characterized. It includes nine members, WW domain-containing protein 1 and 2 (WWP1 and WWP2), NEDD4-1 and NEDD4-2, Smad ubiquitination regulatory factor 1 and 2 (Smurf1 and Smurf2), ITCH, and NEDD4-like 1 and 2 (NEDL1 and NEDL2) that share common structural features (5). All members contain an N-terminal C2 domain responsible for membrane localization, two to four WW domains that mediates protein-protein interaction through PPxY motifs, and a C-terminal catalytic HECT domain (5).

Here, we investigate the regulation of WWP1, a versatile E3 that ubiquitinates and regulates the stability, activity, or cellular localization of several proteins crucial for cancer progression such as transforming growth factor-beta (TGF-ß) type I receptor (6, 7), PTEN (8, 9, 10), LATS1 (11), KLF5 (12), or the members of the p53 superfamily proteins DNp63a, TAp63a, and DNp73 (13). Most of these substrates are implicated in cell proliferation, cell survival, and apoptosis, attributing a role to WWP1 in these processes (11, 13, 14, 15). Moreover, WWP1 dysregulation is associated with tumor progression. *WWP1* expression is significantly increased in breast cancer and various other types of cancer, as evidenced by its gene amplification or mRNA overexpression, and high expression of *WWP1* predicts poor prognosis (14, 16, 17, 18, 19). Therefore, a tight regulation of WWP1 E3 ubiquitin activity is required to maintain cellular homeostasis. A well-described regulatory mechanism of WWP1, shared with all the members of the NEDD4 family, is driven by autoinhibitory intramolecular interactions. Members of the NEDD4 family adopt a closed inactive conformation by interaction of its HECT domain with the C2 domain, WW domains and/or a linker region between the WW domains, thereby blocking access to the HECT catalytic site (20, 21, 22). These autoinhibitory interactions can be relieved by phosphorylation or through the binding of proteins containing PPxY motifs that bind to the WW domains, resulting in enhanced catalytic E3 ligase (23, 24, 25). Several PPxY containing proteins such as Nedd4 family-interacting proteins (NDFIPs) and arrestin-domain containing proteins (ARRDCs) have been shown to interact with the WW domains of NEDD4 E3s and to act either as direct targets, regulators, or adaptors for substrates. In the case of WWP1, Smad7 has been reported to act as an adaptor by enhancing the binding of WWP1 to TβRI (TGF-β type I receptor), thereby inducing TβRI and degradation leading to a global inhibition of the TGF-β signaling pathway (6, 7).

In this study, we identified the putative membrane protein cysteine and tyrosine-rich protein 1 (CYYR1) of unknown function (26) as a novel PPxY-containing protein that binds to WWP1 and to the related NEDD4 family E3s WWP2 and ITCH. We show that CYYR1 localizes to Rab7-labeled late endosomes and interacts with WWP1 in a PPxY-WW dependent manner to induce WWP1 K63-linked autoubiquitination. CYYR1 overexpression decreases WWP1 protein levels, and this effect is abolished by PPxY deletion or lysosome inhibition, indicating that CYYR1 triggers lysosomal degradation of WWP1. Moreover, by analyzing CYYR1 interactome, we identified ANKyrin repeat domain-containing protein 13 A (ANKRD13A) as a partner of CYYR1. We show that ANKRD13A forms a complex with CYYR1 and polyubiquitinated WWP1 and is required for CYYR1-induced degradation of WWP1. Finally, we found that CYYR1 attenuates anchorage-dependent and independent colony formation of breast cancer cells. Moreover, the expression of *CYYR1* is decreased in breast cancer samples and low *CYYR1* expression is associated with poor prognosis. Taken together, our results unprecedentedly highlight a potential protective role of CYYR1 in breast cancer tumorigenesis that could be attributed to its ability to promote WWP1 autoubiquitination and degradation.

## Results

### CYYR1 interacts with WWP1 and WWP2 E3 ubiquitin ligases

**Figure 1:**
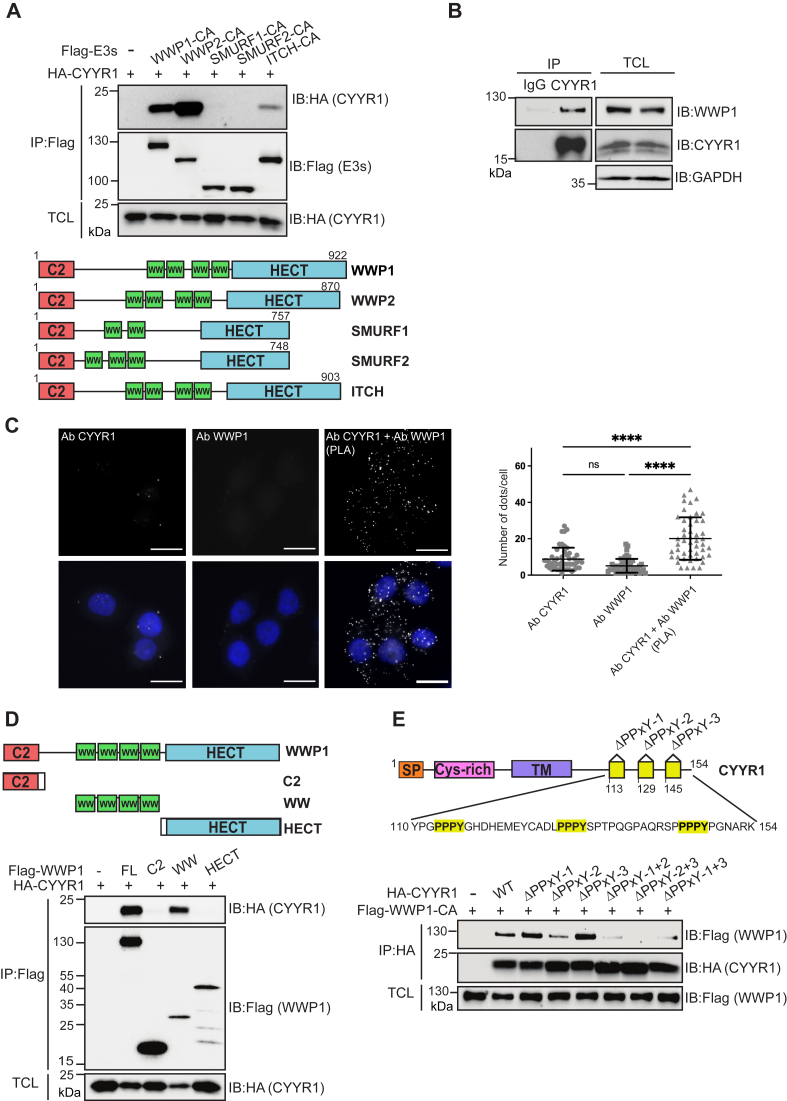
**CYYR1 interacts with WWP1 E3 ubiquitin ligases.***A*, association of CYYR1 with NEDD4 family E3s. HEK293 cells were transfected with HA-CYYR1 either alone or with Flag-tagged catalytically inactive E3s as indicated. Cell lysates immunoprecipitated with anti-Flag antibody and total cell lysates (TCLs) were analyzed by Western blotting with the indicated antibodies. A schematic representation of the NEED4 family E3s showing the C2, WW, and HECT domain. *B*, association between endogenous CYYR1 and WWP1. MDA-MB-468 total cell lysate immunoprecipitated or not with anti-CYYR1 or IgG antibody were analyzed by Western blotting as indicated. *C*, proximity of CYYR1 and WWP1. Proximity ligation assay (PLA) experiments were performed in MDA-MB-468 cells in presence of the indicated antibodies. Antibodies alone conditions are used as a negative control. Dapi staining is shown in the *lower* panel. The bar represents 10 μm. Statistical analysis of the number of dots/cell was performed on 50 cells of one representative experiment using one-way ANOVA followed by Sidak’s test. *D*, schematic representation of the WWP1 constructs showing the C2, WW, and HECT domains. *Lower* panel: WWP1 binding domain to CYYR1. HEK293 cells were transfected with HA-CYYR1 either alone or with full length or domains of Flag-WWP1, as indicated. Cell lysates immunoprecipitated with anti-Flag antibody were analyzed as in A. *E*, schematic representation of the CYYR1 constructs. PS (peptide signal), CYS-Rich (cystein-rich domain), TM (transmembrane domain) and PPxY motifs 1 to 3 are represented. *Lowe*r panel: CYYR1 binding domain to WWP1. HEK293 cells were transfected with Flag-WWP1-CA either alone or with HA-CYYR1-ΔPPxY mutants as indicated. Cell lysates immunoprecipitated with anti-HA antibody were analyzed as described. CA, catalytically inactive; CYYR1, cysteine and tyrosine-rich protein 1; HA, hemagglutinin; HECT, homologous to E6AP C terminus; NEDD4, neural precursor cell-expressed developmentally downregulated 4; WWP1, WW domain-containing protein 1.

To search for regulators of WWP1, we performed a yeast two-hybrid screen using WWP1 as bait and identified CYYR1 as a positive hit. CYYR1 is a putative membrane protein that only had evidence of existence at transcriptional level (27, 28). The presence of three PPxY motifs at the putative C-terminal cytoplasmic tail of the protein sequence suggested a possible function as a regulator of WWP1. To test the specificity of this interaction we performed Flag coimmunoprecipitation experiments in HEK293 cells cotransfected with hemagglutinin (HA)-tagged CYYR1 and Flag-tagged catalytically inactive (CA) mutants E3s of the NEDD4 family (Flag-WWP1-CA, Flag-WWP2-CA, Flag-ITCH-CA, Flag-SMURF1-CA, and Flag-SMURF2-CA) (Fig. 1*A*). We observed that CYYR1 indeed interacts with WWP1, but also with WWP2 and to a lesser extent with ITCH, which are both the most closely related members of WWP1 in the NEDD4 family. No interaction with SMURF1 or SMURF2 was detected, suggesting a specificity of interaction of CYYR1 with WWP1, WWP2, and ITCH.

Because the endogenous CYYR1 protein has not been observed previously, we next sought to validate this interaction in mammalian cells. Since WWP1 is frequently overexpressed in breast cancer we searched for breast cancer cell lines that express CYYR1 mRNA in the databases (https://www.proteinatlas.org). While we noticed that very few breast cancer cell lines express CYYR1 mRNA, we were able to confirm its expression in MDA-MB-468 cell lysates by Western blotting using a commercially available CYYR1 antibody that we validated by depleting the cells of CYYR1 with two independent siRNAs (Fig. S1*A*). We found that this CYYR1 antibody immunoprecipitates endogenous CYYR1 and coimmunoprecipitates endogenous WWP1 (Fig. 1*B*) and WWP2 (Fig. S1*B*) in MDA-MB-468 cell lysates. Moreover, we performed proximity ligation assays (PLAs) using antibodies targeting CYYR1 and WWP1 in MDA-MB-468 cells that enabled the detection of a proximity between endogenous CYYR1 and WWP1, as visualized by the fluorescent dots observed only in the presence of both antibodies (Fig. 1*C*). We also observed proximity of CYYR1 and WWP2 in similar experiments using CYYR1 and WWP2 antibodies (Fig. S1*C*).

We characterized the interaction of CYYR1 with WWP1 and WWP2 by expressing HA-CYYR1 and Flag-tagged constructs containing the different domains of WWP1 and WWP2 in HEK293 cells, and immunoprecipitating the complex with the Flag antibody. As predicted, we observed that the WW domains of WWP1 and WWP2 are sufficient for CYYR1 binding (Figs. 1*D* and S1*D*). Because CYYR1 presents three PPxY motifs, we investigated more precisely which PPxY motif of CYYR1 is involved in this binding by generating HA-tagged CYYR1 mutants lacking one or two PPxY motifs (HA-CYYR1-ΔPPxY). Coimmunoprecipitation experiments of these mutants with Flag-WWP1-CA indicated that while deletion of one PPxY has barely any effect, pairwise PPxY deletions (mainly ΔPPxY-2+3) drastically abolished the binding between CYYR1 and WWP1 and WWP2 (Figs. 1*E* and S1*E*). Altogether these results demonstrate that CYYR1 is a novel protein that interacts with the WW domains of WWP1 and WWP2 through multiple PPxY motifs.

### CYYR1 is ubiquitinated by WWP1 and WWP2 E3 ubiquitin ligases

**Figure 2:**
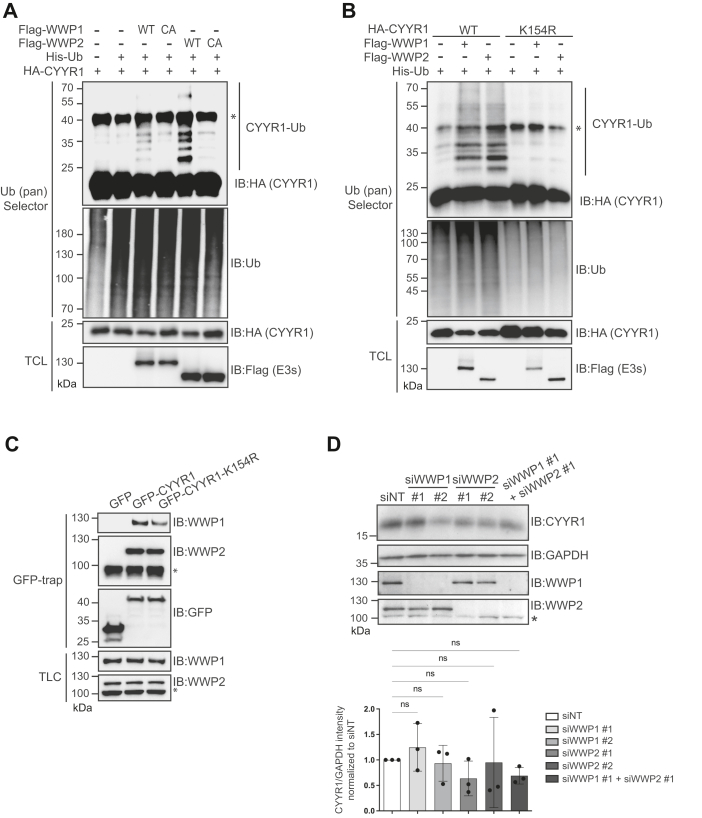
**CYYR1 is ubiquitinated by WWP1 and WWP2 at lysine K154.***A* and *B*, CYYR1 is ubiquitinated by WWP1 and WWP2 at lysine K154. HEK293 cells were transfected with HA-CYYR1-WT or HA-CYYR1-K154R either alone or with His-Ubiquitin (His-Ub) and Flag-tagged WWP1 or WWP2 (WT) or their catalytically inactive mutants (CA) and treated with MG132 for 4 h before lysis. Cell lysates were pulled-down with the ubiquitin pan Selector affinity resin and analyzed by Western blotting with the indicated antibodies. Western blotting on TCL is shown as a transfection control. *C*, CYYR1-K154R binds to WWP1 and WWP2. Cell lysates from HEK293 cells transfected with GFP-CYYR1 or GFP-CYYR1-K154R were immunoprecipitated with the GFP-trap affinity resin and analyzed by Western blotting as indicated. *D*, MDA-MB-468 cells were transfected with a nontargeted siRNA control (siNT) or two independent siRNA (#1 and #2) targeting WWP1 or WWP2. Seventy-two hours posttransfection, cell lysates were analyzed by Western blotting. Quantifications of the CYYR1 intensity relative to GAPDH intensity in each condition normalized to the siNT control condition and *p*-values were calculated using one-way ANOVA followed by Dunnett’s test (n = 3). ∗ on anti-WWP2 Western blot indicates nonspecific band. CYYR1, cysteine and tyrosine-rich protein 1; HA, hemagglutinin; TCL, total cell lysate; WWP1/2, WW domain-containing protein 1/2.

To investigate the possibility that CYYR1 is a substrate for WWP1 and WWP2 E3 ubiquitin ligases, we immunoprecipitated ubiquitinated proteins from lysates of HEK293 cells transfected with HA-CYYR1 and Flag-WWP1 or WWP2 using the ubiquitin pan Selector affinity resin, and observed polyubiquitination of HA-CYYR1 in the presence of Flag-WWP1-WT or Flag-WWP2-WT, but not in the presence of their CA mutant (Fig. 2*A*). To identify which lysine residue of CYYR1 was the target of WWP1- or WWP2-mediated ubiquitination, we generated lysine-to-arginine substitution mutants of CYYR1 for each of the five lysines presents on the CYYR1 protein (K16R, K33-36R, K89R, and K154R). A dramatic decrease of CYYR1 polyubiquitination induced by WWP1 or WWP2 was observed with the CYYR1-K154R mutant (Figs. 2*B* and S2) although this mutant has retained its ability to bind both E3s (Fig. 2*C*). These results indicate that WWP1 and WWP2 mediate CYYR1 polyubiquitination on lysine 154. To evaluate the effect of WWP1 and WWP2 on CYYR1 protein level, we next depleted WWP1 or/and WWP2 in MDA-MB-468 cells, but could not detect any effect on CYYR1 stability at endogenous level (Fig. 2*D*). This could be explained either by the fact that WWP1/2 does not ubiquitinate CYYR1 under these endogenous conditions, or because WWP1/2 induces nondegradative ubiquitination of CYYR1. Since CYYR1 does not exhibit known function, we could not conclude on the consequences of WWP1/2-mediated ubiquitination on CYYR1. We therefore next investigated whether CYYR1 could function as a novel regulator for WWP1/2.

### CYYR1 regulates WWP1 autoubiquitination and protein level

**Figure 3:**
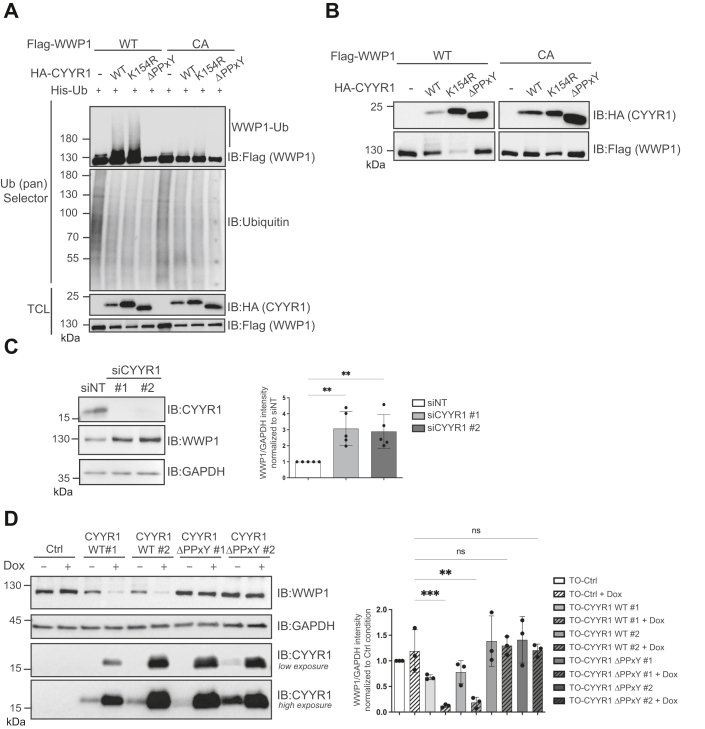
**CYYR1 regulates WWP1 autoubiquitination and protein level.***A*, CYYR1 increases WWP1 autoubiquitination. HEK293 cells transfected with His-Ub and Flag-WWP1-WT or Flag-WWP1-CA either alone or with different HA-CYYR1 constructs as indicated. Protein cell lysates were pulled-down with the ubiquitin pan Selector affinity resin and analyzed by Western blotting. *B*, CYYR1 decreases WWP1 protein level. Cell lysates form HEK293 cells transfected with Flag-WWP1-WT or Flag-WWP1-CA either alone or with different constructs for HA-CYYR1 were analyzed by Western blotting. *C*, CYYR1 depletion increases WWP1 protein level. Cell lysates from MDA-MB-468 cells transfected with a nontargeting siRNA control (siNT) or two independent siRNA (#1 or #2) targeting CYYR1 were analyzed by Western blotting using the indicated antibodies. Quantifications of the WWP1 intensity relative to GAPDH in each condition were normalized to the siNT control condition, and *p*-values were calculated with a one-way ANOVA followed by Dunnett’s test (n = 5). *D*, CYYR1 decreases endogenous WWP1 protein level. MDA-MB-231 cells expressing doxycycline (Dox)-inducible CYYR1-WT or CYYR1-ΔPPxY (TO-CYYR1-WT or TO-CYYR1-ΔPPxY clones #1 and #2) were treated with Dox for 24 h before analysis of the cell lysates by Western blotting. Low and high exposure of the anti-CYYR1 Western blot are shown to highlight CYYR1 expression leakiness in absence of Dox. Quantifications of the WWP1 intensity relative to GAPDH in each condition were normalized to the Ctrl condition and *p*-values were calculated with one-way ANOVA followed by Dunnett’s test (n = 3). CYYR1, cysteine and tyrosine-rich protein 1; Dox, doxycycline; WWP1, WW domain-containing protein 1.

Since HECT E3 ligases from the NEDD4 family are known to interact with PPxY-containing proteins to relieve their autoinhibitory conformation, we investigated whether CYYR1 might have a similar regulatory effect on WWP1 autoubiquitination. We coexpressed different mutants of HA-CYYR1 and Flag-WWP1 in HEK293 cells and purified ubiquitinated proteins with the ubiquitin pan Selector resin to evaluate the ubiquitination level of WWP1 (Fig. 3*A*). We observed that CYYR1-WT increases the polyubiquitination of WWP1-WT, but not of WWP1-CA that is catalytically inactive, indicating that CYYR1 induces WWP1 polyubiquitination through its intrinsic E3 ligase activity and does not involve another endogenous E3. This ubiquitination was slightly enhanced in the presence of CYYR1-K154R, presumably due to the absence of concomitant CYYR1 ubiquitination of this mutant but was not observed with CYYR1-ΔPPxY2+3 (renamed CYYR1-ΔPPxY throughout this study) that could not interact with WWP1. Autoubiquitination of WWP1 and WWP2 has been reported to be associated with protein instability (6, 21, 29). We therefore assessed the effect of CYYR1 on WWP1 protein level. First, we noticed that HA-CYYR1 reduces the protein level of Flag-WWP1-WT but not of Flag-WWP1-CA coexpressed in HEK293 cells (Fig. 3*B*). Moreover, the HA-CYYR1-K154R mutant which lacks ubiquitination site, enhanced this reduction, whereas the HA-CYYR1-ΔPPxY mutant showed no effect, which was consistent with their respective effects on WWP1 ubiquitination (Fig. 3*A*).

We then evaluated the effect of CYYR1 depletion on WWP1 protein level in the MDA-MB-468 cell line that expresses endogenous CYYR1. SiRNA targeting of CYYR1 in this cell line induced an increase of WWP1 protein level (Fig. 3*C*) and we confirmed by quantitative reverse transcription polymerase chain reaction (RT-qPCR) that this increase was not due to transcriptional upregulation of WWP1 (Fig. S3*A*). Conversely, we sought to stably express CYYR1 in MDA-MB-231, a breast cancer cell line that does not express CYYR1. We generated two doxycycline (Dox)-inducible MDA-MB-231 cell lines that express untagged CYYR1 protein after Dox treatment. We compared the protein level of WWP1 in MDA-MB-231 expressing the empty Dox-inducible vector (TO-Ctrl) and two MDA-MB-231 clones that express Dox-inducible CYYR1-WT (clones TO-CYYR1-WT#1 and TO-CYYR1-WT#2) or CYYR1-ΔPPxY (clones TO-CYYR1-ΔPPxY#1 and TO-CYYR1-ΔPPxY#2). Corroborating our findings, the endogenous WWP1 protein was significantly decreased after induction of CYYR1-WT expression in both clones whereas expression of CYYR1-ΔPPxY had no impact on WWP1 protein level (Fig. 3*D*). Interestingly, we noticed that due to the leakiness of the Tet-Operator inducible system (as shown by the high exposure of CYYR1 Western blot), WWP1 protein level is already reduced in the absence of Dox treatment, indicating a CYYR1 dose response for WWP1 regulation. As previously, we confirmed by RT-qPCR that this diminution is not due to a reduction of WWP1 mRNA (Fig. S3*B*).

Altogether, these results indicate that endogenous CYYR1 regulates WWP1 ubiquitination and protein level. We performed the equivalent experiments with WWP2 and noticed that overexpression of CYYR1 also induced WWP2 ubiquitination and protein level decrease (Fig. S4, *A*–*C*). Intriguingly CYYR1 depletion in MDA-MB-468 had no effect on WWP2 protein level (Fig. S4*D*). We conclude that endogenous CYYR1 protein preferentially regulates WWP1 stability in MDA-MB-468 cells.

### CYYR1 induces a lysosome-mediated WWP1 degradation

**Figure 4:**
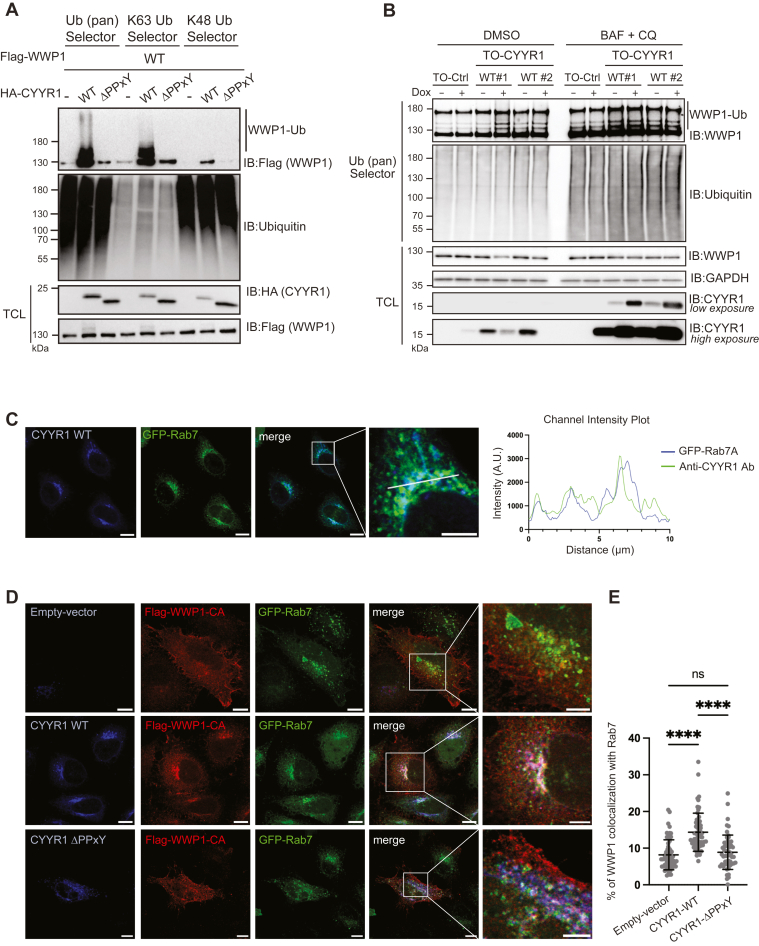
**CYYR1 induces lysosome-dependent degradation of WWP1.***A*, CYYR1 induces K63-linked polyubiquitination of WWP1. HEK293 cells were transfected with Flag-WWP1-WT either alone or with different constructs for HA-CYYR1 as indicated. Cell lysates were pulled-down with the ubiquitin pan, the ubiquitin K63 or the ubiquitin K48 Selector affinity resins and analyzed by Western blotting using the indicated antibodies. *B*, CYYR1 induces polyubiquitination of endogenous WWP1. MDA-MB-231 TO-Ctrl or TO-CYYR1-WT clones #1 and #2 were treated with Dox 10 ng/ml and with bafilomycin A1 (BAF) 100 nM and chloroquine (CQ) 50 μM or DMSO for 24 h before lysis. Cell lysates were pulled-down with the ubiquitin pan Selector affinity resins and analyzed by Western blotting using the indicated antibodies. *C*, CYYR1 colocalizes with GFP-Rab7A. HeLa cells transfected with untagged CYYR1 and GFP-Rab7A (*green*) were stained by immunofluorescence with anti-CYYR1 antibody (*blue*). Channel intensity plots along a 10 μm line is shown. *D*, WWP1 colocalizes with CYYR1 at GFP-Rab7A-tagged late endosomes. HeLa cells transfected with Flag-WWP1-CA and GFP-Rab7A (*green*) in presence or in absence of untagged CYYR1-WT or CYYR1-ΔPPxY, were subjected to immunofluorescence with anti-CYYR1 (*blue*) and anti-Flag (*red*). *E*, CYYR1 targets WWP1 to GFP-Rab7A-tagged late endosomes. The % of colocalization of Flag-WWP1-CA with GFP-Rab7A was measured in each condition on 20 cells in three independent experiments. This percentage was determined by measuring the intensity of anti-Flag staining within the perinuclear GFP-Rab7-stained area relative to the overall anti-Flag staining intensity of each cell. Statistical analysis was performed with one-way ANOVA followed by Sidak’s test. The scale bar in each image represents 10 μm. The scale bar in the enlarged image represents 5 μm. CYYR1, cysteine and tyrosine-rich protein 1; DMSO, dimethyl sulfoxide; Dox, doxycycline.

To investigate the mechanism by which CYYR1 regulates WWP1 protein level, we sought to determine which polyubiquitination linkage of WWP1 is induced by CYYR1. Using ubiquitin Selector affinity resins that recognize either the K48 linkage or the K63 linkage, we found that CYYR1 clearly induces selective K63-linked autoubiquitination of WWP1 in a PPxY-dependent manner (Fig. 4*A*), indicating that CYYR1 might induce lysosomal degradation of WWP1. Importantly, we also observed increased polyubiquitination of endogenous WWP1 in response to CYYR1 expression in the MDA-MB-231 clones, which is concomitant with WWP1 degradation (Fig. 4*B*, left panel). Moreover, lysosome inhibition with bafilomycin A1 and chloroquine treatment attenuates CYYR1-mediated WWP1 degradation in the MDA-MB-231 clones (Figs. 4*B*, right panel and S5) and leads to accumulation of endogenous polyubiquitinated WWP1 (Fig. 4*B*, right panel).

Since CYYR1 is a novel protein that harbors a transmembrane domain, we therefore set out to determine its cellular localization by immunofluorescence experiments. We evaluated the colocalization of untagged CYYR1 coexpressed with different GFP-tagged membrane trafficking protein markers (data not shown) and discovered that CYYR1 colocalizes with GFP-tagged Rab7A, a marker of late endosomes (30) (Fig. 4*C*). We then analyzed the colocalization of Flag-WWP1-CA with CYYR1-WT or the CYYR1-ΔPPxY mutant. Consistently, we observed colocalization of Flag-WWP1-CA with CYYR1-WT, whereas colocalization is significantly reduced with CYYR1-ΔPPxY mutant that lacks the PPxY interaction motifs for WWP1 binding (Fig. 4, *D* and *E*). Moreover, while Flag-WWP1-CA is distributed throughout the cell in the absence of CYYR1 or in the presence of CYYR1-ΔPPxY, its coexpression with CYYR1-WT leads to a significant accumulation of WWP1 at late endosomes marked with GFP-Rab7A. These observations suggest that CYYR1 recruits cytoplasmic WWP1 at the surface of late endosome. Since late endosomes are known to fuse to lysosomes (31, 32), this recruitments might contribute to WWP1 trafficking toward lysosomal degradation.

### ANKRD13A is involved in CYYR1-mediated WWP1 degradation

**Figure 5:**
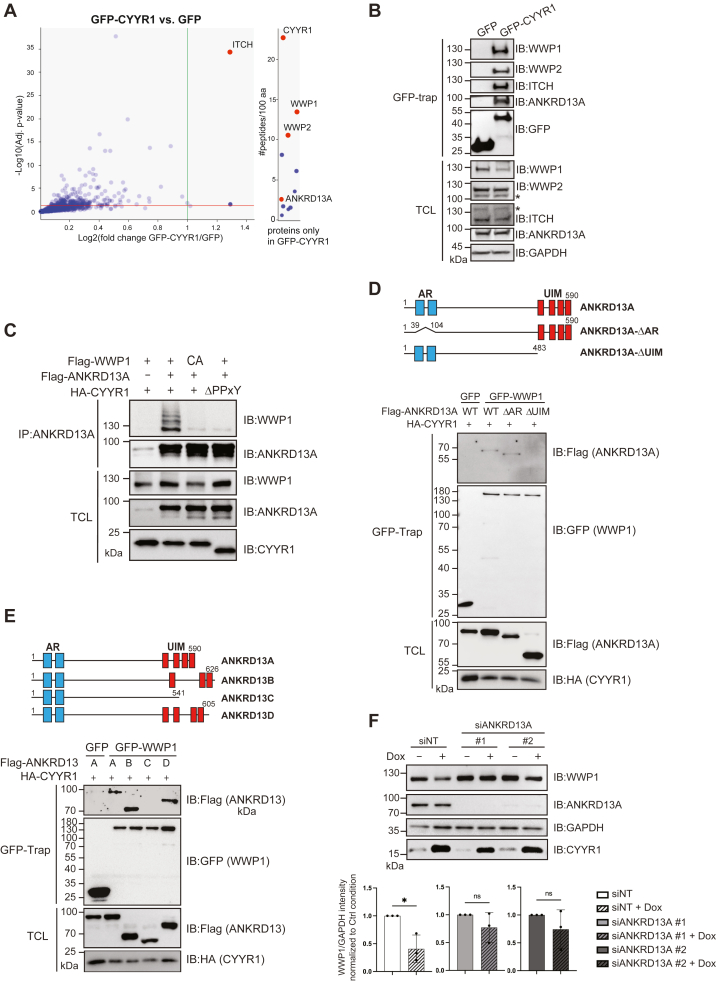
**ANKRD13A is involved in CYYR1-induced WWP1 degradation.***A*, CYYR1-interactome. Cell lysates from HEK293 cells transfected with GFP or GFP-CYYR1 were purified on GFP-Trap affinity resin and analyzed by quantitative label-free mass spectrometry. Volcano plot showing differentially expressed proteins significantly enriched in GFP-CYYR1 compared to GFP (*green line* correspond to fold change ≥ 2, and red line to *p*-value ≤ 0.05, n = 5) that display at least three peptides in each of the five replicate experiments. Proteins that display no common peptides in the GFP condition (infinite ratio) are represented on the *left* of the graph with their distribution based on the number of peptides identified/100aa. *B*, CYYR1 interacts with ANKRD13A. Cell lysates from HEK293 cells transfected with GFP or GFP-CYYR1 were purified with GFP-Trap affinity resin and analyzed by Western blotting with the indicated antibodies. *C*, WWP1 interacts with ANKRD13A in the presence of CYYR1. HEK293 cells were transfected with Flag-WWP1 or Flag-WWP1-CA and HA-CYYR1-WT or HA-CYYR1-ΔPPxY with or without Flag-ANKRD13A. Cell lysates were immunoprecipitated with anti-ANKRD13A antibody and immunoblotted with the indicated antibodies. *D*, schematic representation of the ANKRD13A constructs. AR and UIM domains are shown. *Lower* panel: ANKRD13A binds WWP1 through its UIM domains. HEK293 cells were transfected with HA-CYYR1, Flag-ANKRD13A-WT, or deletion mutants and GFP or GFP-WWP1-WT as indicated. Cell lysates were immunoprecipitated with the GFP-trap affinity resin and immunoblotted with the corresponding antibodies. *E*, schematic representation of the ANKRD13A, *B*, *C*, and *D*. *Lower panel*: WWP1 binds all members of the ANKRD13 family except ANKRD13C. Cell lysates from HEK293 cells were transfected with HA-CYYR1 and Flag-tagged ANKRD13 family proteins, and GFP or GFP-WWP1-WT were immunoprecipitated with the GFP-trap affinity resin and analyzed by Western blotting with the indicated antibodies. *F*, ANKRD13A depletion attenuates the degradation of WWP1 induced by CYYR1. MDA-MB-231 TO-CYYR1-WT clone #1 were transfected with control siRNA (siNT) or two independent siRNA targeting ANKRD13A. Seventy-two hours post transfection, cells were treated with 500 pg/ml Dox for 24 h before lysis and Western blotting analysis with the indicated antibodies. Quantifications of the WWP1 intensity relative to GAPDH in each + Dox condition were normalized to the -Dox condition and *p*-values were calculated by performing a paired *t* test, ∗*p* < 0.05 (n = 3). ANKRD13A, ANKyrin repeat domain-containing protein 13 A; AR, ankyrin-repeat; CYYR1, cysteine and tyrosine-rich protein 1; Dox, doxycycline; UIM, ubiquitin-interacting motif; WWP1, WW domain-containing protein 1.

To get further insight into the mechanism of action of CYYR1-mediated WWP1 degradation, we analyzed the interactome of CYYR1. We performed GFP-Trap affinity purification of GFP-CYYR1 or GFP individually expressed in HEK293 cells followed by quantitative label-free mass spectrometry (MS), and compared the interactomes of GFP-CYYR1 to GFP (Fig. 5*A* and Table S1). WWP1, WWP2, and ITCH were among the most robust hits, which confirmed that CYYR1 specifically regulates WWP1 and the related E3 WWP2 and ITCH. Interestingly, we also detected the protein ANKRD13A, a protein known to interact with K63-linked ubiquitinated proteins to regulate their endolysosomal trafficking toward lysosomal degradation (33, 34).

We confirmed ANKRD13A binding to CYYR1 by Western blot analysis of the GFP copurified proteins lysates (Fig. 5*B*), using a commercially available ANKRD13A antibody that we had previously validated by siRNA targeting (Fig. S6*A*). We further identified CYYR1 and ANKRD13A interaction at endogenous level by detecting coimmunoprecipitation of ANKRD13A with CYYR1 in MDA-MB-468 cells (Fig. S6*B*). We then evaluated WWP1 binding to the CYYR1-ANKRD13A complex by co- ANKRD13A immunoprecipitation experiments in HEK293 cells transfected with Flag-ANKRD13A and different mutants of WWP1 and CYYR1 (Fig. 5*C*). We found that ANKRD13A coimmunoprecipitates higher molecular weight forms of Flag-WWP1-WT in the presence of HA-CYYR1-WT but not in the presence of HA-CYYR1-ΔPPxY, and does not coimmunoprecipitate Flag-WWP1-CA in the presence of HA-CYYR1-WT. These observations indicate that ANKRD13A interacts with WWP1 specifically under conditions where WWP1 undergoes autoubiquitination in the presence of CYYR1. Interestingly, endogenous ANKRD13A is known to interact with K63-linked ubiquitinated protein *via* multiple ubiquitin-interacting motifs (UIMs) localized in its C-terminal region (33, 34). We therefore evaluated their involvement in ANKRD13A-WWP1 binding by generating the ANKRD13A-ΔUIM mutant as well as the ANKRD13A-ΔAR mutant deleted of the N-terminal ankyrin-repeat (AR) domain (Fig. 5*D*). GFP-Trap purification of GFP-WWP1 indicated that WWP1 binds ANKRD13A-WT or the ANKRD13A-ΔAR mutant in the presence of CYYR1 but not the ANKRD13A-ΔUIM mutant, demonstrating that polyubiquitinated WWP1 binds to ANKRD13A through the UIM motifs (Fig. 5*D*). The ANKRD13 family contains three other members ANKRD13B, C, and D. Noteworthy, ANKRD13C does not exhibit any UIMs and we found that WWP1 redundantly binds each member of the ANKRD13 family except ANKRD13C (Fig. 5*E*). Finally, we observed that the depletion of ANKRD13A by two independent siRNA significantly attenuates the degradation of WWP1 induced by CYYR1 expression upon Dox treatment in MDA-MB-231 TO-CYYR1-WT clones (Fig. 5*F*). Altogether, these results indicate that ANKRD13A interacts with K63-polyubiquitinated WWP1 bound to CYYR1 and is involved in CYYR1-mediated targeting of WWP1 toward lysosomal degradation.

### CYYR1 expression limits anchorage-dependent and independent colony formation of MDA-MB-231 breast cancer cells

**Figure 6:**
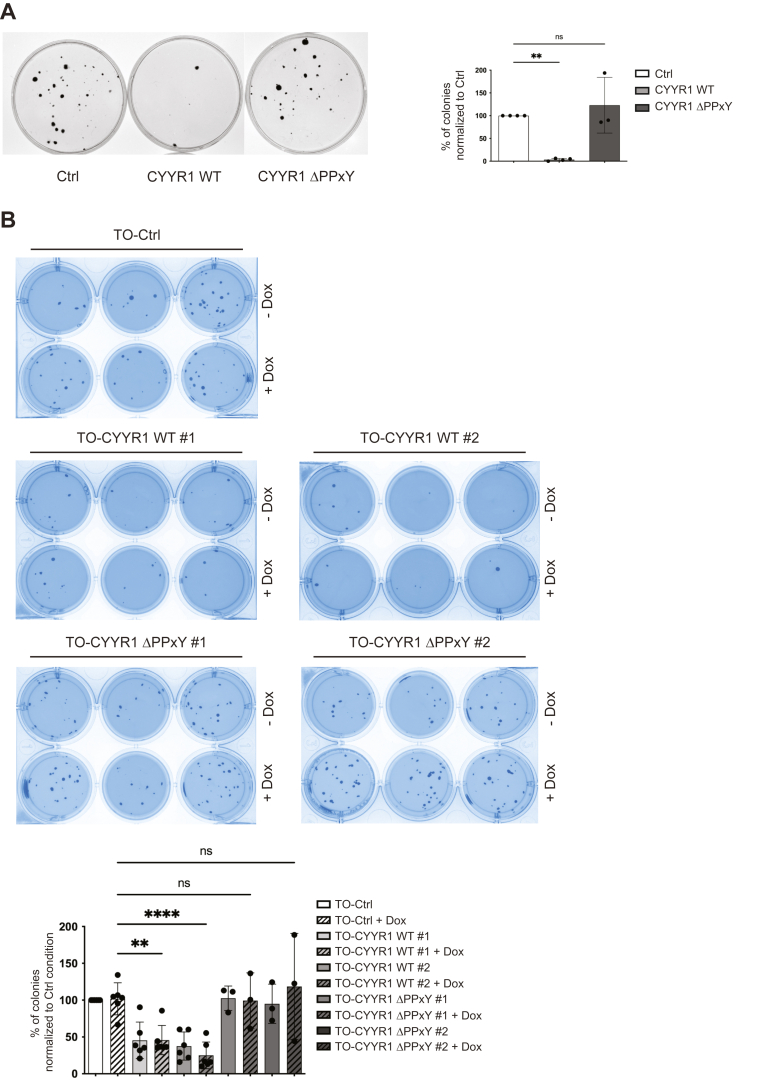
**CYYR1 expression limits anchorage-dependent and independent colony formation of MDA-MB-231 breast cancer cells.***A*, MDA-MB-231 cells were transfected with pMEP4-CYYR1-WT or pMEP4-CYYR1-ΔPPxY before selection with hygromycin B. Colonies were stained with crystal violet and counted. Statistical analysis was performed with one-way ANOVA followed by Dunnett’s test (Ctrl *versus* CYYR1-WT, n = 4; Ctrl *versus* CYYR1-ΔPPxY, n = 3). *B*, MDA-MB-231 TO-Ctrl, TO-CYYR1-WT or -ΔPPxY clones were plated for soft agar growth assay in presence or absence of doxycycline. Colonies were stained with Thiazolyl Blue Tetrazolium Bromide coloration and counted. The % of colonies obtained in each condition relative to the TO-Ctrl condition are represented (Ctrl *versus* CYYR1-WT, n = 6 independent experiments; Ctrl *versus* CYYR1-ΔPPxY, n = 3). Statistical analysis was performed with one-way ANOVA followed by Dunnett’s test. CYYR1, cysteine and tyrosine-rich protein 1.

WWP1 is overexpressed in cancer and has been shown to increase cell proliferation and cell survival (35). Having established a role of CYYR1 in WWP1 degradation, we then set out to explore the role of this novel protein in these cellular processes related to cancer progression. While we could not detect a significant role of CYYR1 in cell proliferation (data not shown), we provide evidence that CYYR1 limits cell growth in anchorage-dependent and independent colony formation, while WWP1 has been shown to promote cell growth in similar assays (8, 11, 14, 36). First, we found that MDA-MB-231 expressing CYYR1-WT failed to grow into colonies on solid surface, whereas cells expressing CYYR1-ΔPPxY showed the same ability as the control cells expressing the empty vector (Fig. 6*A*). Moreover, we performed colony formation assay in soft agar by using the two MDA-MB-231 TO-CYYR1-WT clones, and found that the expression of CYYR1 inhibits anchorage-independent cell growth as evidenced by the significant decrease of the number of colonies compared to control cells expressing the empty vector (Fig. 6*B*). This effect was independent of the addition of Dox, indicating that the leakiness of CYYR1 expression in the absence of Dox observed by Western blot (Fig. 3*D*) is sufficient to induce this effect. Importantly, we did not observe any significant decrease of colony formation in soft agar assay performed on the two MDA-MB-231 TO-CYYR1-ΔPPxY clones expressing CYYR1-ΔPPxY. Altogether, these results indicate that CYYR1 expression limits anchorage-dependent and independent colony formation of MDA-MB-231 breast cancer cell and that this effect is dependent on its PPxY motifs.

### *CYYR1* expression is decreased in breast tumors and is associated with beneficial clinical outcome

**Figure 7:**
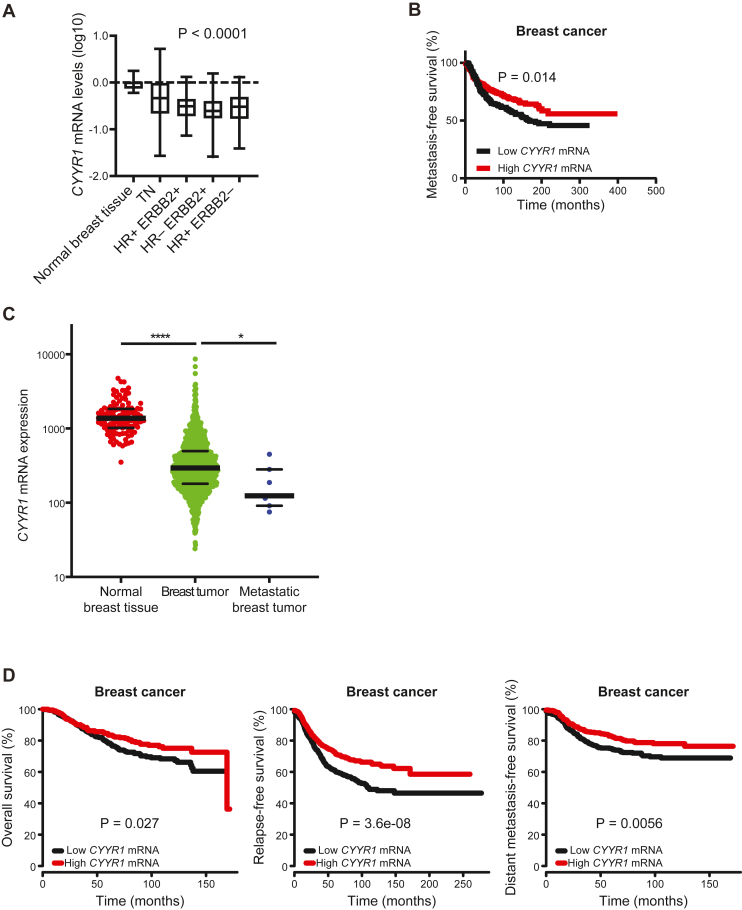
***CYYR1* expression is decreased in breast cancer and associated with beneficial clinical outcome.***A*, analysis of the mRNA level of *CYYR1* by RT-qPCR on normal breast tissue and four different human breast tumor subtypes, triple-negative breast cancer (TN; HR-ERBB2-), hormone receptor positive/HER2 positive (HR + ERBB2+), hormone receptor negative/HER2 positive (HR-ERBB2+) and hormone receptor positive/HER2 negative (HR + ERBB2-) in a cohort of 505 breast cancer patients. *p*-value was calculated using the Kruskal-Wallis H test. *B*, Kaplan-Meier analysis of metastasis-free survival rates of patients with tumors expressing high (*red line*) *versus* low (*black line*) levels of *CYYR1* mRNA expression in the same cohort of 505 breast cancer patients. *C*, mRNA level of *CYYR1* from the KM plotter database in normal breast tissues, breast tumor tissues and metastatic breast tumor tissues. *D*, Kaplan-Meier analyses of overall survival, relapse-free survival or distant metastasis-free survival according to *CYYR1* mRNA expression in KM plotter database of breast cancer. CYYR1, cysteine and tyrosine-rich protein 1; RT-qPCR, quantitative reverse transcription polymerase chain reaction.

Based on our novel findings, we predicted that *CYYR1* expression might be downregulated in human breast tumors in comparison to normal breast tissue. We first determined mRNA levels of *CYYR1* in a large cohort of 505 human breast tumors from patients with well-documented follow-up by RT-qPCR. Strikingly, *CYYR1* mRNA levels were strongly decreased in breast tumors, irrespective of the molecular subtype, in comparison to normal breast tissue (Fig. 7*A*). Then, we evaluated in the same cohort the prognostic value of *CYYR1* on patient metastasis-free survival stratifying patient samples into groups of low and high expression of *CYYR1*. This revealed that patients with low expression of *CYYR1* had the poorest prognosis (Fig. 7*B*). Finally, we observed similar results on *CYYR1* by interrogating the publicly available human cancer KM Plotter database that contains gene expression data and survival information. Indeed, *CYYR1* mRNA levels were decreased in breast primary tumors and even more when the primary tumor has metastasized (Fig. 7*C*). In addition, a low expression of *CYYR1* was associated with poor prognosis when considering overall survival, relapse-free survival or distant metastasis-free survival (Fig. 7*D*). Taken together, these data unambiguously show that *CYYR1* is associated with beneficial clinical outcome in breast tumors.

## Discussion

**Figure 8:**
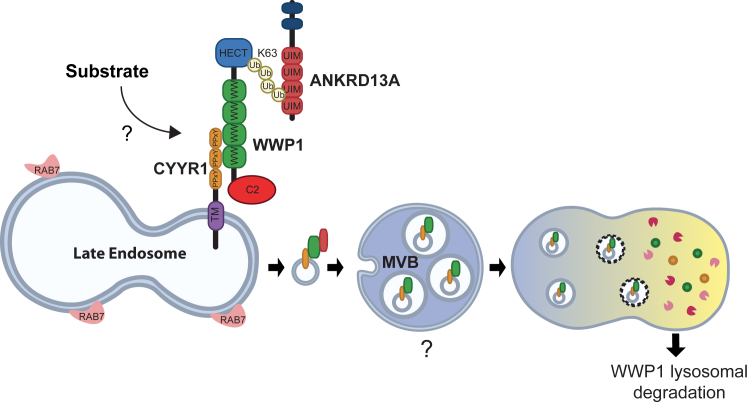
**Model for lysosome degradation of WWP1 mediated by CYYR1.** CYYR1 localizes at late endosomes where it recruits WWP1 in a PPxY/WW dependent manner. This interaction triggers K63-linked auto-ubiquitination of WWP1 that allows subsequent binding of ANKRD13A in a Ubiquitin/UIM dependent manner. These interactions targets WWP1 toward lysosomal degradation resulting in an overall decrease of WWP1 protein level. ANKRD13A, ANKyrin repeat domain-containing protein 13 A; CYYR1, cysteine and tyrosine-rich protein 1; UIM, ubiquitin-interacting motif; WWP1, WW domain-containing protein 1.

In this study, we report a new mechanism of regulation for the E3 ubiquitin ligase WWP1 that involves the novel protein CYYR1. We showed that CYYR1 localizes at late endosomes where it binds to WWP1 in a PPxY/WW dependent manner. This interaction leads to K63-linked autopolyubiquitination of WWP1 that subsequently allows binding of ANKRD13A in a ubiquitin/UIM-dependent manner. These interactions drive WWP1 toward lysosomal degradation resulting in a decrease of WWP1 protein level (Fig. 8).

Among the nine members of the Nedd4 E3 ligases family that all contains WW domains, we found that CYYR1 preferentially interacts with WWP1, WWP2, and ITCH. This specificity is supported by the exclusive identification of these three E3s in the CYYR1 interactome that corroborates the lack of interaction observed between CYYR1 and Smurf1/2 in immunoprecipitation experiments. Interestingly, WWP1, WWP2, and ITCH share a common architectural structure of their WW domains, consisting of four consecutive WW domains and a crucial linker region positioned between the second and third WW domains (20, 21). Additional work is needed to understand how this distinctive structural feature of the WW domains drives specificity of interaction with the multiple PPxY motifs of CYYR1. The binding preference of CYYR1 suggests that its ability to induce degradation of WWP1 might also extend to WWP2 and ITCH. However, while CYYR1 overexpression can also induce WWP2 autoubiquitination and degradation, we could not observe any significant effect of CYYR1 depletion on WWP2 protein level in MDA-MB-468 cells. In light of this observation, we propose that CYYR1 may preferentially regulates the stability of WWP1 depending of its expression level in the cell.

We demonstrate that CYYR1 interaction with WWP1 leads to autoubiquitination of WWP1 but also to ubiquitination of CYYR1 on lysine 154, which suggests that CYYR1 acts as a regulator but could also constitute a novel substrate of WWP1. Since we could not provide evidence of an increased stability of endogenous CYYR1 in WWP1/2-depleted MDA-MB-468 cells, the consequences of WWP1/2-mediated ubiquitination on CYYR1 remains to be clarified. One possibility could be that nondegradative ubiquitination of CYYR1 is required to induce WWP1 lysosomal degradation. However, we found that the K154R mutant can still induce the ubiquitination (Fig. 3*A*) and degradation (Fig. 3*B*) of WWP1, indicating that it is not the case. Whether WWP1-ubiquitination of CYYR1 mediated by WWP1 could regulate unknown CYYR1 functions need to be investigated in a future study.

CYYR1 contains an N-terminal peptide signal, a central cysteine-rich domain followed by a transmembrane protein and a C-terminal region containing three successive PPxY motifs (26). We found that CYYR1 localizes at late endosomes labeled by Rab7A and interacts with the WW domains of WWP1 through its PPxY motifs. It is therefore likely that CYYR1 localizes at the membrane of the late endosome and catches cytoplasmic WWP1 at the surface of the late endosome through its PPxY-containing C-terminal tail. Interestingly proteins of the NDFIP (NDFIP1 and 2) and the ARRDC (ARRDC1 to 5) families that also contain PPxY motifs and localize at the endolysosomal compartment have been shown to recruit NEDD4-1/2, ITCH, and WWP2, and to a less extent WWP1, in order to ubiquitinate and induce lysosomal degradation of cargo proteins such as the receptor CXCR4 (37), Robo1 (24), and GPCR (38). Here, we demonstrate that WWP1 is also recruited to late endosomes by the CYYR1 protein that belongs to the STMC6 family. This family of proteins that includes four other members in mammals, WBP1, VOPP1, WBP1L/OPAL, and TMEM92, also contains a transmembrane domain and multiple PPxY motifs (26). Interestingly WBP1L/OPAL1 has been shown to act as an adaptor of WWP1, WWP2, ITCH, and NEDD4L at the plasma membrane for CXCR4 ubiquitination and degradation (39). In light of these studies and our finding, it is therefore likely that the STMC6 proteins might also constitute an important family of proteins that regulates NEDD4-like E3s, and more particularly WWP1.

NDFIP1/2, ARRDC1-5, and other PPxY containing proteins that bind to the WW domains of the NEDD4 family E3s, have been shown to act as adaptors for substrates or as direct substrates (25). Since their binding alleviates the autoinhibitory intramolecular interaction within the E3, these adaptors have also been shown to induce additional autoubiquitination of the E3 (20, 39). Moreover mutations in the WW-linker or the HECT domain of WWP1/2 that also lead to an open conformation have been shown to promote uncontrolled autoubiquitination (21, 22). In this study, we have demonstrated that CYYR1 acts as a direct regulator of WWP1 that induces WWP1 K63 autoubiquitination and lysosomal degradation. Whether CYYR1 induces a change in the targeted lysine(s) ubiquitinated on WWP1 that could help its recruitment to lysosomes needs to be clarified in future study.

However, we could not exclude that CYYR1 could also act as an adaptor for the recruitment of unknown substrates for WWP1-mediated ubiquitination at the endosomal surface, prior to drive WWP1 lysosomal degradation. Such substrates might be found within the list of our candidates identified in the CYYR1 interactome, and future work will be required to explore this possibility. Nonetheless, our analysis of the top 10 candidates of the CYYR1 interactome, led us to identify the protein ANKRD13A as part of the CYYR1-WWP1 complex. ANKRD13A has been shown to localize on the endolysosomal compartment and to trigger lysosomal degradation of K63-ubiquitinated caveolin 1 or EGF receptor in a UIM-dependent manner (33, 34). Our study reveals that it is also required for CYYR1-mediated lysosomal degradation of K63-ubiquitinated WWP1, which strengthen the role of ANKRD13A in the endolysosomal trafficking of K63-ubiquitinated specific targets. Since we found that WWP1 can also bind ANKRD13B, ANKRD13D, two other ANKRD13 family members that contain UIM motifs, it would be interesting in the future to evaluate the functional redundancy or specificity of these different ANKRD13 proteins in CYYR1-mediated WWP1 degradation.

Because WWP1 has been associated with breast and prostate cancer progression, we sought to investigate the role of CYYR1 in cancer. We found that CYYR1 impairs cell growth in anchorage-dependent and independent colony formation assays. Deletion of the PPxY motifs abolishes this effect, indicating that CYYR1-WWP1 interaction might be required. Since WWP1 depletion has been shown to decrease cell growth of breast and prostate cancer cells in similar assays (8, 11, 14, 36), we speculate that the effect of CYYR1 on cell growth may be mediated by WWP1 degradation. Alternatively, since we found that WWP1 ubiquitinates CYYR1 on its lysine 154 in a PPxY dependent manner, another possibility could be that CYYR1 ubiquitination triggers its function through a yet uncharacterized mechanism. Moreover, we could not exclude the possibility that upon particular cellular condition, WWP1-mediated ubiquitination of CYYR1 could induce CYYR1 degradation. The relevance of CYYR1 ubiquitination at lysine K154 in its cellular function will need to be addressed in future work.

Most importantly, our study identified a potential protective role in cancer for CYYR1. Indeed, analysis of *CYYR1* expression in our breast cancer cohort and in the KM plotter database, both reveal that *CYYR1* expression is significantly decreased in breast cancer samples compared to normal breast tissue. Moreover, low *CYYR1* expression correlates with a poor prognosis with a higher risk of relapse or to develop distant metastasis. Conversely, increase of *WWP1* expression in breast cancer has been associated with poor prognosis (18, 19, 40). Collectively, these observations suggest that *CYYR1* downregulation in breast cancer could favor tumor progression by increasing WWP1 protein.

## Experimental procedures

### Yeast two-hybrid screen

The yeast two-hybrid screen was performed by Hybrigenics. Complementary DNA (cDNA) encoding for human full-length WWP1 was cloned into pB27 as a C-terminal fusion to LexA (N-LexA-WWP1-C fusion). The resulting plasmid was used as bait in yeast two-hybrid screens of a placental cDNA library. By screening 10 x 10^6^ colonies of a human placental cDNA library, we obtained 13 different cDNA species, one of which encodes CYYR1.

### Cell culture, cells transfections, and establishment of stables cells lines

MDA-MB-468 cells were grown at 37 °C in 100% air, in Leibovitz’s L-15 medium supplemented with 10% fetal bovine serum (FBS), glutamine 2 mM, and antibiotics (100 U/ml penicillin and streptomycin). MDA-MB-231, HEK293 and HeLa cells were grown at 37 °C in 5% CO_2_ in Dulbecco's modified Eagle's medium GlutaMAX supplemented with 10% FBS and antibiotics (100 U/ml penicillin and streptomycin). Transient transfections of plasmids were performed using X-tremeGENE HP (Sigma-Aldrich) according to manufacturer’s instructions, and the cells were incubated for 24 h in complete media. Dox-inducible stable cell lines (Tet-Rex system, Thermo Fisher Scientific) were generated by a first round of selection of MDA-MB-231 cells transfected with pCDNA6-TR (Thermo Fisher Scientific) using blasticidin (15 μg/ml). The clone with the most efficient expression of the Tet repressor was subsequently selected with hygromycin B (100 μg/ml) after transfection with pCDNA5-TO (Thermo Fisher Scientific) or the different pCDNA5-TO-CYYR1 constructs described in the plasmids section. The control MDA-MB-231 TO-Ctrl cells were selected as a pool, while the MDA-MB-231 TO-CYYR1-WT and MDA-MB-231 TO-CYYR1-ΔPPxY were selected as clones by anti-CYYR1 Western blotting, and two clones for each condition were used for further experiments (clones #1 and #2). Stable cell lines were grown at 37 °C in 5% CO_2_ in Dulbecco's modified Eagle's medium GlutaMAX supplemented with 10% “Tet-System Approved” FBS and antibiotics (100 U/ml penicillin and streptomycin). Dox induction was performed at 10 ng/ml for 24 h. siRNA transfections were performed using 50 nM of siRNA and Lipofectamine RNAiMAX (Invitrogen) in complete media according to the manufacturer’s instructions. Cells lysates were harvested 72 h posttransfection. SiRNA targeting CYYR1 (#1: SI04152981, #2: SI04283825), WWP1 (#1: SI04287689, #2: SI04293030), WWP2 (#1: SI03095344, #2: SI04952626), and ANKRD13A (#1: SI04139800, #2: SI04320218), and the nontargeting siRNA control siNT (1027281) were purchased from Qiagen.

### Plasmids and constructions

The following plasmids were previously described: pCMV10-Flag-WWP1-WT, pCMV10-Flag-WWP1-C890A, pCMV10-Flag-WWP1-C2, pCMV10-Flag-WWP1-WW, pCMV10-Flag-WWP1-HECT, pCMV-His-Ub, pCMV7.1-Flag-SMURF1, and Flag-WWP2-WT (6, 41). Flag-tagged CA mutants pCMV5B-Flag-SMURF2-CA (C716A) was a gift from Jeff Wrana (Addgene plasmid # 11747) (42); pCMV7.1-Flag-ITCH-CA (C803A) was described previously (43); Flag-SMURF1-CA (C699A) and Flag-WWP2-CA (C838A), were generated by site-directed mutagenesis using QuickChange Lightning kit (Agilent Technologies, #210519). pEGFP-WWP1 was generated by PCR subcloning of pCMV10-Flag-WWP1-WT in pEGFP. Flag-WWP2 domain constructs (C2, WW, and HECT) were generated by PCR subcloning into pCMV10. GFP-Rab7A was a gift from Gia Voeltz (Addgene plasmid # 61803) (44). HA-CYYR1 expression vector was generated by PCR using the CYYR1_pCSdest plasmid (a gift from Roger Reeves, Addgene plasmid # 53794) (45) as a template and subcloning at BamHI/KpnI in HAHA-pCMV5. HA-CYYR1 mutants (K16R, K33-36R, K89R, and K154R) and deletions (individually or by pair) of its PPxY motifs (ΔPPxY-1 (aa113–116), ΔPPxY-2 (aa129–132), and ΔPPxY-3 (aa145–148)) were generated by site-directed mutagenesis. GFP-CYYR1 expression vectors GFP-CYYR1-WT and GFP-CYYR1-K154R were generated by using HA-CYYR1 WT or HA-CYYR1-K154R as template and subcloning at BamH1/KpnI in pEGFP-C1. Untagged CYYR1 expression vectors pMEP4-CYYR1-WT and pMEP4-CYYR1-ΔPPxY-2+3 were generated by PCR using HA-CYYR1 WT and ΔPPxY-2+3, respectively, as template and subcloned at BamHI/KpnI into pMEP4 (Invitrogene). For generation of Dox-inducible cell lines, pCDNA5-TO-CYYR1-WT and pCDNA5-TO-CYYR1-ΔPPxY#2  + 3 were generated by PCR using pMEP4-CYYR1 WT or pMEP4-CYYR1-ΔPPxY#2  + 3 as template and subcloning at BamHI/KpnI into pCDNA5-TO. Flag-Tagged ANKRD13 family cDNAs cloned in the pcDNA3.1 plasmid (pCDNA3.1+/C-(K)-DYK-ANKRD13) were purchased from GenScript: ANKRD13A (OHu13104); ANKRD13B (OHu06736), ANKRD13C (OHu13704) and ANKRD13D (OHu11812). Flag-ANKRD13A depleted mutants (ΔAR (aa40–103) and ΔUIM (aa484–590)) were generated by site-directed mutagenesis. All constructs were verified by sequencing.

### Reagents and antibodies

For proteasome inhibition: MG-132 10 μM (474790, Sigma-Aldrich), for lysosome inhibition: chloroquine 50 μM (C6628, Sigma-Aldrich) and bafilomycin A1 100 nM (201550, ChemCruz). The following antibodies were used for either Western blotting, immunoprecipitation, or immunofluorescence as indicated: anti-HA (3F10, Roche; 0.1 μg/IP), anti-Flag (M2, Sigma-Aldrich; 1 μg/IP), anti-GFP (6663, Abcam), anti-GAPDH-HRP (47724, Santa Cruz), anti-CYYR1 (HPA067685, Sigma-Aldrich; 1 μg/IP), anti-WWP1 (H00011059-M01, Abnova), anti-WWP2 (12197-1-AP, Proteintech), anti-ITCH (D20,11890, Santa Cruz), anti-Ubiquitin (P4D1, 8017, Santa Cruz), anti-ANKRD13A (23998-1-AP, Proteintech; 1 μg/IP), anti-rabbit-Alexa-Fluor-647 (A21244, Invitrogen).

### Cellular lysis, immunoprecipitation, and Western blotting

For preparation of cellular lysates, cells were harvested after transfection and/or stimulation, and lysed at 4 °C in TNMG buffer (20 mM Tris-HCl pH 8, 150 mM NaCl, 5 mM MgCl_2_, 0.5% NP-40, 10% glycerol) supplemented with 10 mM NaF, 10 mM ß-glycerophosphate and EDTA-free proteases Inhibitor (54925800, Roche) for 15 min, centrifuged at 15,000 rpm for 13 min and supernatants were retained. Protein estimation was performed with BCA protein quantification assay kit (Pierce).

For immunoprecipitation, cells lysates containing 500 μg to 1 mg protein were subjected to immunoprecipitation with the appropriates antibody overnight at 4 °C followed by adsorption to Sepharose-coupled protein G for 1 h at 4 °C. Immunoprecipitates were washed three times with TNMG buffer. Proteins were separated by SDS-PAGE and analyzed by immunoblotting using a standard procedure with the indicated antibodies. Western blot was acquired on a ChemiDoc XRS+ (Bio-Rad) and quantification were performed by measuring the adjust intensity using Image Lab 4.0 (Bio-Rad, https://www.bio-rad.com/fr-fr/product/image-lab-software).

### Ubiquitination assay

For immunoprecipitation of ubiquitinated proteins, cells were treated or not with 10 μM MG132 for 4 h before lysis in radioimmunoprecipitation assay buffer supplemented with 10 mM NaF, 10 mM ß-glycerophosphate, EDTA-free proteases Inhibitor (Roche) and 10 mM N-Ethylmaleimide. The sonicated (10 s ON, 10 s OFF three times) and cleared lysates were immunoprecipitated with 20 μl of pan, K48 or K63 ubiquitin Selector affinity resins (NanoTag Biotechnologies, #N2510, ,#N1810, or #N1910) for 1 h and washed three times with radioimmunoprecipitation assay buffer.

### Mass spectrometry

For GFP-immunoprecipitation (GFP-Trap, ChromoTek Cat# gta-20), HEK293 cells were plated in P100 culture plates and transfected by GFP or GFP-CYYR1. Twenty-four hours posttransfections, cells were lysed in IP150 buffer (20 mM Tris–HCl pH 7.5, 150 mM NaCl, 5 mM EDTA, 1% NP-40, and 10% glycerol) supplemented with 10 mM NaF, 10 mM ß-glycerophosphate, EDTA-free proteases Inhibitor (Roche), and cleared supernatants were retained. Subsequently, 3.5 mg of proteins were incubated on 25 μl of GFP-Trap ChromoTek slurry overnight at 4 °C, followed by three washes with IP150 buffer, 1 wash with IP150 buffer 0,1% NP-40, three washes with ammonium bicarbonate 50 mM buffer. Beads were resuspended in 100 μl of ammonium bicarbonate buffer and proteins digested by adding 0.2 μg of trypsin-LysC (Promega) for 1 h at 37 °C. Samples were then loaded into custom-made C18 StageTips packed by stacking three AttractSPE disk (#SPE-Disks-Bio-C18-100.47.20 Affinisep) into a 200 μl micropipette tip for desalting. Peptides were eluted using a ratio of 40:60 CH3CN:H2O + 0.1% formic acid and vacuum concentrated to dryness with a SpeedVac apparatus. Peptides were reconstituted in 10 μl of injection buffer in 0.3% trifluoroacetic acid before liquid chromatography-tandem mass spectrometry analysis.

Liquid chromatography-tandem mass spectrometry analysis: Online chromatography was performed with an RSLCnano system (Ultimate 3000, Thermo Fisher Scientific) coupled to a Q Exactive HF-X with a Nanospray Flex ion source (Thermo Fisher Scientific). Peptides were first trapped on a C18 column (75 μm inner diameter × 2 cm; nanoViper Acclaim PepMap 100, Thermo Fisher Scientific) with buffer A (2/98 MeCN/H2O in 0.1% formic acid) at a flow rate of 2.5 μl/min over 4 min. Separation was then performed on a 50 cm × 75 μm C18 column (nanoViper Acclaim PepMap RSLC, 2 μm, 100 Å, Thermo Fisher Scientific) regulated to a temperature of 50 °C with a linear gradient of 2% to 30% buffer B (100% MeCN in 0.1% formic acid) at a flow rate of 300 nl/min over 91 min. MS full scans were performed in the ultrahigh-field Orbitrap mass analyzer in ranges m/z 375 to 1500 with a resolution of 120,000 at m/z 200. The top 20 intense ions were isolated and subjected to further fragmentation *via* high energy collision dissociation activation by a resolution of 15,000 with the automatic gain control target set to 1e5 ions. We selected ions with charge state from 2+ to 6+ for screening. Normalized collision energy was set at 27 and the dynamic exclusion of 40s.

Data processing protocol: For identification, the data were searched against the Homo sapiens (UP000005640) UniProt database using Sequest HT through Proteome Discoverer (version 2.4, https://www.thermofisher.com/us/en/home/industrial/mass-spectrometry/liquid-chromatography-mass-spectrometry-lc-ms/lc-ms-software/multi-omics-data-analysis/proteome-discoverer-software.html). Enzyme specificity was set to trypsin and a maximum of two miss cleavages sites were allowed. Oxidized methionine, Met-loss, Met-loss-Acetyl, and N-terminal acetylation were set as variable modifications. Maximum allowed mass deviation was set to 10 ppm for monoisotopic precursor ions and 0.02 Da for MS/MS peaks. The resulting files were further processed using myProMS v3.10.0 (46) (https://github.com/bioinfo-pf-curie/myproms). False discovery rate calculation used Percolator and was set to 1% at the peptide level for the whole study. The label-free quantification was performed by peptide extracted ion chromatograms (XICs), reextracted peptides across the five replicates together from each condition and computed with MassChroQ version 2.2.21 (http://pappso.inrae.fr/bioinfo/masschroq/) (47). For protein quantification, XICs from proteotypic peptides shared between compared conditions (TopN matching) and missed cleavages were allowed. Median and scale normalization was applied on the total signal to correct the XICs for each biological replicate (N = 5 in each conditions). To estimate the significance of the change in protein abundance, a linear model (adjusted on peptides and biological replicates) was performed, and *p*-values were adjusted using the Benjamini–Hochberg false discovery rate procedure.

The MS proteomics raw data have been deposited to the ProteomeXchange Consortium *via* the PRIDE (48) partner repository with the dataset identifier PXD050895.

### Immunofluorescence

HeLa cells plated on coverslips were fixed with 4% paraformaldehyde and blocked and permeabilized with 0.3% Triton X-100 3% bovine serum albumin in PBS for 1 h at room temperature. Cells were incubated overnight at 4 °C with the indicating antibody or for 1 h at room temperature when using Alexa Fluor-conjugated antibodies and secondary antibodies. Washings were performed in PBS-0.1% Triton X-100 and DNA staining was performed with Dapi. Coverslips were then mounted in Prolong diamond antifade reagent (Life Technologies) and visualized on confocal Olympus Fluoview 300 inverted microscope. Image analysis was performed using the Fiji software (https://imagej.net/software/fiji/).

### Proximity ligation assay

PLA was performed using the Duolink *In Situ* PLA Kit (Sigma-Aldrich). MDA-MB-468 cells were plated on coverslips and fixed as described above for immunofluorescence assays. Coverslips were incubated with anti-CYYR1 and anti-WWP1 or WWP2 antibodies for 45 min at room temperature, washed with PBS and incubated with secondary antibodies coupled with DNA probes (Duolink *In Situ* PLA Anti-Mouse Plus and Anti-Rabbit Minus antibodies, Sigma-Aldrich) for 1 h. This was followed by incubation with the Duolink *In Situ* Detection Reagents Orange (Sigma-Aldrich) following the manufacturer’s instructions for DNA probes hybridization and circularization, PCR amplification, and fluorescent staining of the amplified DNA. PCR amplification only occurs when both antibodies are in close proximity (<40 nm) and protein proximity is visualized as florescent dots. Control coverslips were treated with only one primary antibody to estimate background staining in each experiment. Coverslips were stained with Dapi, mounted in Prolong diamond antifade reagent and analyzed on a Leica DM4B microscope.

### Anchorage-dependent and independent colony formation assays

For anchorage-dependent colony formation assay, MDA-MB-231 were seeded in P60 plates at 80,000 cells/ml. After 24 h, cells were transfected by 4 μg of pMEP4-CYYR1 WT or mutants or the empty pMEP4 plasmid as control. Twenty-four hours posttransfection, transfected cells were selected by 100 μg/ml hygromycin during 2 weeks. On the last day, all of the plates were fixed in 10% acetic acid and 10% methanol, stained with crystal violet 0.4% and 20% ethanol and counted.

Anchorage-independent colony formation assay (soft agar growth assay) was performed in 12-wells plates on a base layer of 0.6% agar mixed with 5% “Tet-system Approved” FBS growth medium. MDA-MB-231 TO-Ctrl and TO-CYYR1 clones were seeded at 300 cells/well in 0,3% agar mixed with 5% “Tet-system Approved” FBS growth medium in presence or absence of 100 ng/ml Dox. Plates were then incubated under standard culture conditions for 3 weeks allowing for colonies formation. Colonies were colored by addition of 0.5 mg/ml thiazolyl blue tetrazolium bromide and counted.

### Statistical analysis

All experiments were performed at least as three independent biological replicates (n ≥ 3) and quantified when indicated. The means ± SD were calculated and statistical analyses were performed as indicated, either with paired *t* test or with one-way ANOVA followed by Dunnett’s or Sidak’s test using GraphPad Prism software (https://www.graphpad.com) (∗*p* < 0.05, ∗∗*p* < 0.01, ∗∗∗*p* < 0.001, ∗∗∗∗*p* < 0.0001, ns: not significant).

### Breast cancer cohort analysis

Primary breast tumors were obtained from 505 women treated at Institut Curie - Hôpital René Huguenin (Saint-Cloud, France) between 1978 and 2008. Clinical data of the patients and characteristics of the tumors are reported in Table S2. All patients have given their approval for the potential use of their tumor samples for scientific purpose. This study was approved by the local ethics committee (Breast Group of Institut Curie - René Huguenin Hospital) and complies with the principles of the Declaration of Helsinki. The samples were immediately stored in liquid nitrogen until RNA extraction. A tumor sample was considered suitable for this study if the proportion of tumor cells exceeded 70%. All patients (mean age 60.9 years, range 29–91 years) met the following criteria: primary unilateral nonmetastatic breast carcinoma for which complete clinicopathological data and follow-up were available; no radiotherapy or chemotherapy before surgery; and full follow-up at Institut Curie - Hôpital René Huguenin. Estrogen receptor (ER), progesterone receptor (PR), and human epidermal growth factor receptor 2 (ERBB2) statuses were determined at the protein level by biochemical methods (dextran-coated charcoal method, enzyme immunoassay, or immunohistochemistry) and confirmed by real-time quantitative RT-PCR. The population was divided into four groups according to hormone receptors (HR) (ER and PR) and ERBB2 statuses as follows: two luminal subtypes [HR+ (ERα+ or PR+)/ERBB2+ (n = 53)] and [HR+ (ERα+ or PR+)/ERBB2- (n = 288)]; an ERBB2+ subtype [HR- (ERα- and PR-)/ERBB2+ (n = 68)] and a triple-negative subtype [HR- (ERα- and PR-)/ERBB2- (n = 96)]. During a median follow-up of 8.9 years (range 1 month to 33.2 years), 203 patients developed metastasis. Thirteen samples of adjacent normal breast tissue from breast cancer patients and normal breast tissue from women undergoing cosmetic breast surgery were used as sources of normal RNA.

For RT-qPCR on the samples of the breast cancer cohort, total RNA extraction, cDNA synthesis, and RT-qPCR reaction have been described elsewhere (49). Quantitative values were obtained from the cycle number (Ct value) using QuantStudio 7 Flex real-time PCR system (Applied Biosystems). Data from each sample were normalized on the basis of its content in *TBP* transcripts. *TBP* encoding the TATA box-binding protein (a component of the DNA-binding protein complex TFIID) was selected as an endogenous control due to the moderate level of its transcripts and the absence of known *TBP* retro-pseudogenes (retro-pseudogenes lead to coamplification of contaminating genomic DNA and thus interfere with RT-PCR transcripts, despite the use of primers in separate exons). Results, expressed as N-fold differences in *CYYR1* gene expression relative to the *TBP* gene and termed “N_*CYYR1*_”, were determined as N_*CYYR1*_ = 2^ΔCtsample^, where the ΔCt value of the sample was determined by subtracting the average Ct value of *CYYR1* gene from the average Ct value of *TBP* gene. Primers for *CYYR1* (upper primer, 5′- CGTCTCCTCCTATCCTGGACCAC-3′; lower primer, 5′-GGAGGAGGCAAGTCTGCACAGT-3′) and *TBP* (upper primer, 5′-TGCACAGGAGCCAAGAGTGAA-3′; lower primer, 5′-CACATCACAGCTCCCCACCA-3′), were selected with Oligo 6.0 program (National Biosciences, Plymouth, MN, https://www.oligo.net/).

Relationships between mRNA *CYYR1* levels and breast tumor subtypes were identified by using the nonparametric test, namely the Kruskal–Wallis H test (relationship between one quantitative parameter and two or more qualitative parameters). Differences were considered significant at confidence levels greater than 95% (*p* < 0.05).

To visualize the efficacy of a molecular marker to discriminate between two populations (patients that developed/did not develop metastases) in the absence of an arbitrary cutoff value, data were summarized in a receiver operating characteristic curve (50). The area under curve was calculated as a single measure to discriminate efficacy.

Metastasis-free survival was determined as the interval between initial diagnosis and detection of the first metastasis. Survival distributions were estimated by the Kaplan–Meier method, and the significance of differences between survival rates were ascertained with the log-rank test.

### KM plotter database analyses

Data of *CYYR1* mRNA expression from normal breast tissue and breast tumors were extracted from the KM plotter database (https://kmplot.com/analysis/). Analysis of Kaplan–Meier curves evaluating the prognosis value of *CYYR1* mRNA expression was performed with the dataset Affymetrix ID 228665_at with autoselection of the best cut off.

## Data availability

The mass spectrometry proteomics data have been deposited to the ProteomeXchange Consortium *via* the PRIDE partner repository identified with the data set identifier PXD050895.

## Supporting information

This article contains supporting information.

## Conflict of interest

The authors declare that they have no conflicts of interest with the contents of this article.
