# Supplementary material for: CYYR1 promotes the degradation of the E3 ubiquitin ligase WWP1 and is associated with favorable prognosis in breast cancer
Source: J Biol Chem. 2024 Jul 24;300(9):107601. doi: 10.1016/j.jbc.2024.107601 (PMC11399591; doi:10.1016/j.jbc.2024.107601)
Supplement: Supporting Information [file mmc1.pdf]

SUPPORTING INFORMATIONS

CYYR1 promotes the degradation of the E3 ubiquitin ligase WWP1 and is associated with favorable prognosis in breast cancer

Tiphaine Perron, Mathieu Boissan, Ivan Bièche, Laura Courtois, Florent Dingli, Damarys Loew, Mouna Chouchène, Sabrina Colasse, Laurence Levy, Céline Prunier

Figure S1: CYYR1 interacts with WWP2

Figure S2: CYYR1 is ubiquitinated by WWP1 and WWP2 at lysine K154

Figure S3: CYYR1 does not affect WWP1 mRNA level

Figure S4: CYYR1 regulates WWP2 auto-ubiquitination and protein level

Figure S5: Lysosome inhibition attenuates WWP1 degradation induced by CYYR1

Figure S6: CYYR1 interacts with ANKRD13A at endogenous level

Table S1: List of the differentially enriched proteins in GFP-CYYR1 compared to GFP.

Table S2: Histopathological and clinical characteristics of 505 breast cancer patients

Supplementary Figure Perron et al

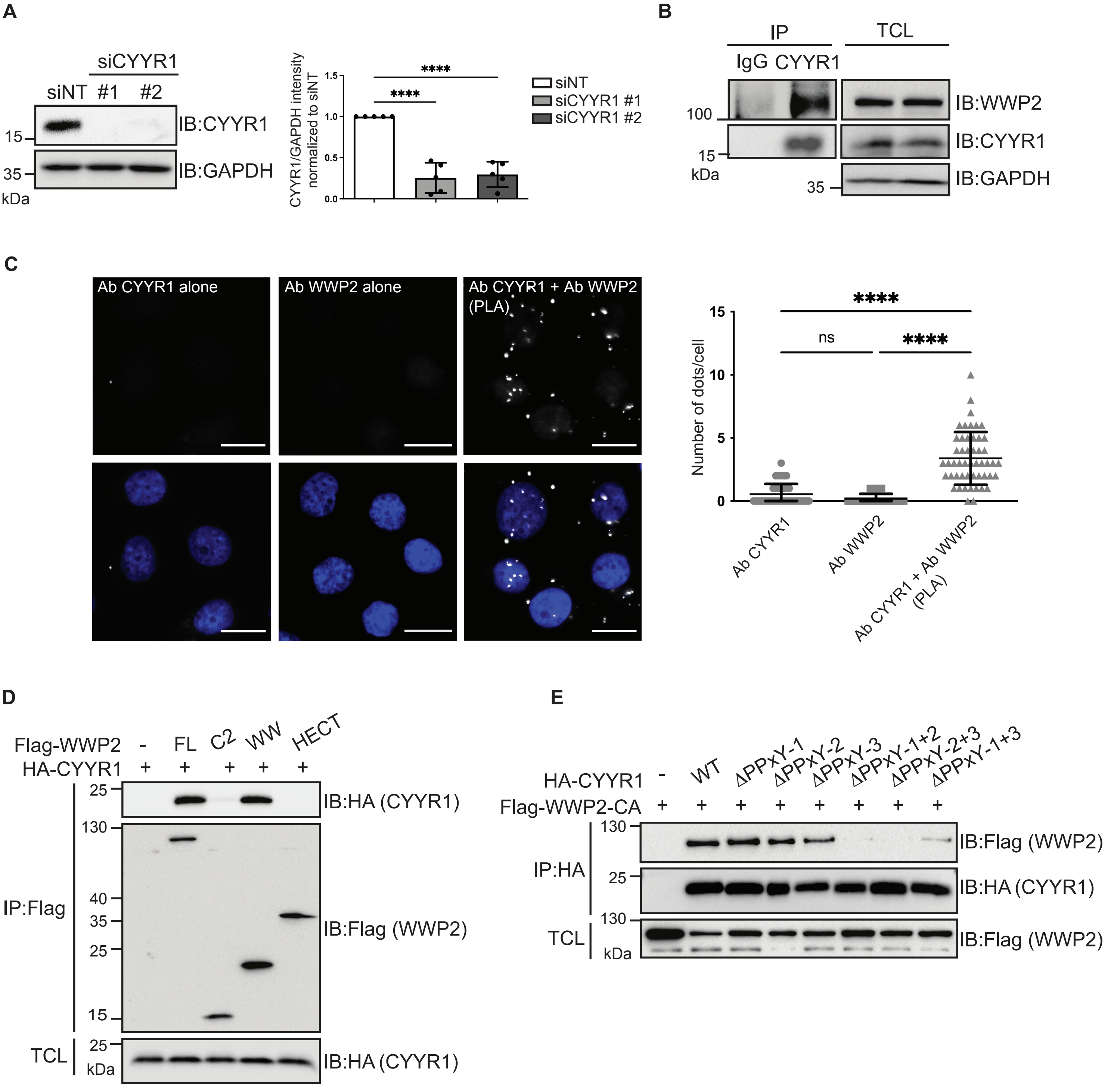

Figure S1: CYYR1 interacts with WWP2

(A) CYYR1 antibody validation. Protein lysates from MDA-MB-468 cells transfected with a non-target siRNA (siNT) or two independent siRNA targgeting CYYR1 were analyzed by western-blot with CYYR1 antibody and GAPDH as a loading control. Statistical analysis was performed with one-way ANOVA followed by Dunnett's test on 5 independent experiments. **(B)** CYYR1 interacts with WWP2. MDA-MB-468 cell lysate immunoprecipitated with anti-CYYR1 or IgG antibody were analyzed by western-blotting as indicated. Western-blotting on total cell lysates (TCL) is shown as a loading control. **(C)** Proximity of CYYR1 and WWP2. Proximity Ligation Assay (PLA) experiments were performed in MDA-MB-468 cells in presence of the indicated antibodies. Dapi staining is shown in the lower panel. Bar: 10 µm. Statistical analysis of the number of dots/cell was performed on 50 cells of one representative experiment using one-way ANOVA followed by Sidak's test. **(D)** WWP2 Binding domain to CYYR1. HEK293 cells were transfected with HA-CYYR1 either alone or with full length or domains of Flag-WWP2, as indicated. Cell lysates immunoprecipitated with anti-Flag antibody were analyzed by western-blotting using anti-Flag or anti-HA antibody. Western-blotting on TCL with anti-HA antibody is shown as a transfection control. **(E)** CYYR1 binding domain to WWP1. HEK293 cells were transfected with Flag-WWP2-CA either alone or with HA-CYYR1-ΔPPxY mutants, as indicated. Cell lysates immunoprecipitated with anti-HA antibody and TCL were analyzed by western-blotting as indicated.

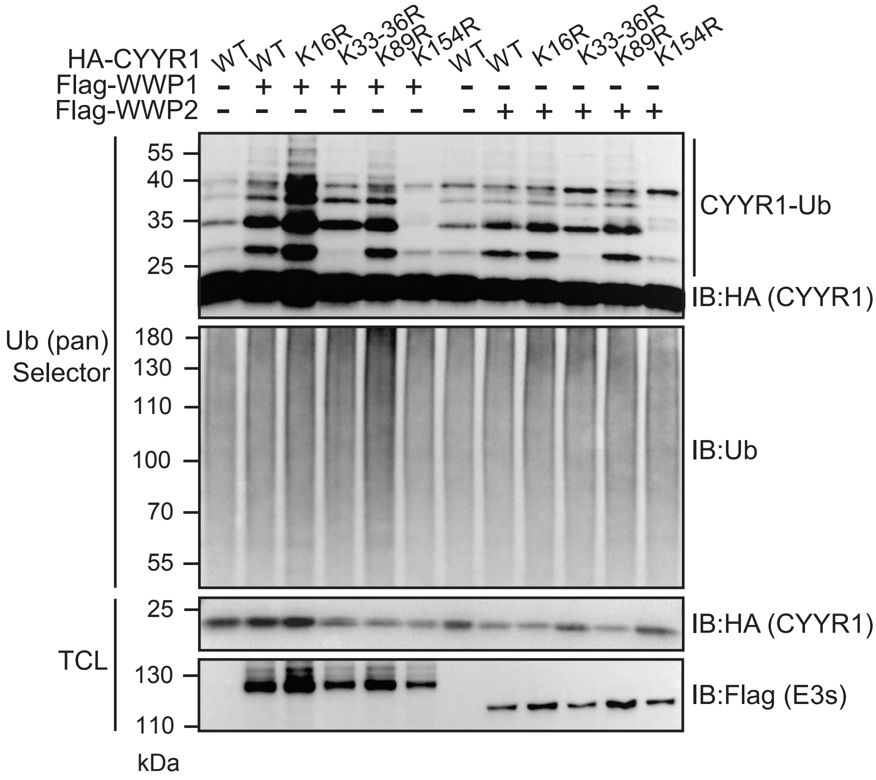

Figure S2: CYYR1 is ubiquitinated by WWP1/2 at lysine K154

HEK293 cells were transfected with HA-CYYR1 wild-type (WT) or HA-CYYR1 KR mutants as indicated, either alone or with Flag-WWP1 or Flag-WWP2. Cell lysates were pulled-down with ubiquitin pan Selector affinity resin and and analyzed by western-blotting.

A

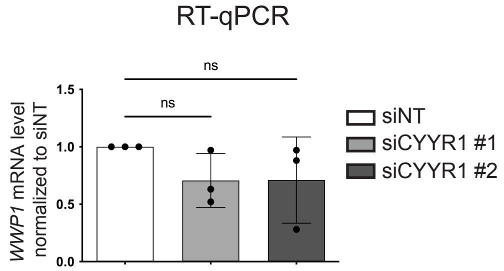

B

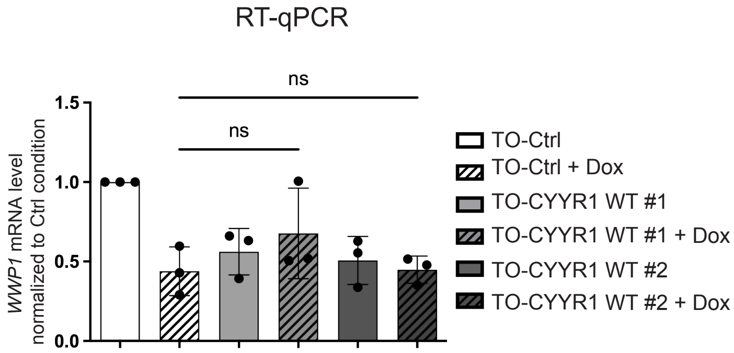

Figure S3: CYYR1 does not affect WWP1 mRNA level
(A) RT-qPCR analysis of *WWP1* mRNA level in MDA-MB-468 transfected with the non-targeting control siRNA (siNT) or two independent siRNA targetting *CYYR1* (siCYYR1#1 and #2).
cDNA were synthetized using the iScript cDNA synthesis kit (Bio-Rad) from RNA extracted with Trizol (Invitrogene).
qPCR was performed in triplicate on *WWP1* (primers WWP1-F TGGCATAGCACAAACTGGTG, WWP1-R GTCCTTGCTGAAGGCTCTCC) and *GAPDH* (primers GAPDH-F TGCACCACCAACTGCTTAGC, GAPDH-R GGCATGGACTGTGGTCATGAG), using SYBR Green qPCR master mix (Biotools) according to manufacturer's protocol in Quant Studio thermocycler (Fisher Scientific).Expression of *WWP1* was calculated by the $2^{-\Delta\Delta Ct}$ method using *GAPDH* as control. All data represent mean ± SD for at least three independent experiments, and p-value were calculated with one-way ANOVA followed by Dunnett's test.
(B) RT-qPCR analysis for *WWP1* mRNA level in the MDA-MB-231 control cells (TO-Ctrl) and the two MDA-MB-231 TO-CYYR1-WT clones #1 and #2, after 24h treatment or not with 10 ng/mL doxycycline.
RT-qPCR analysis was performed as described in A.

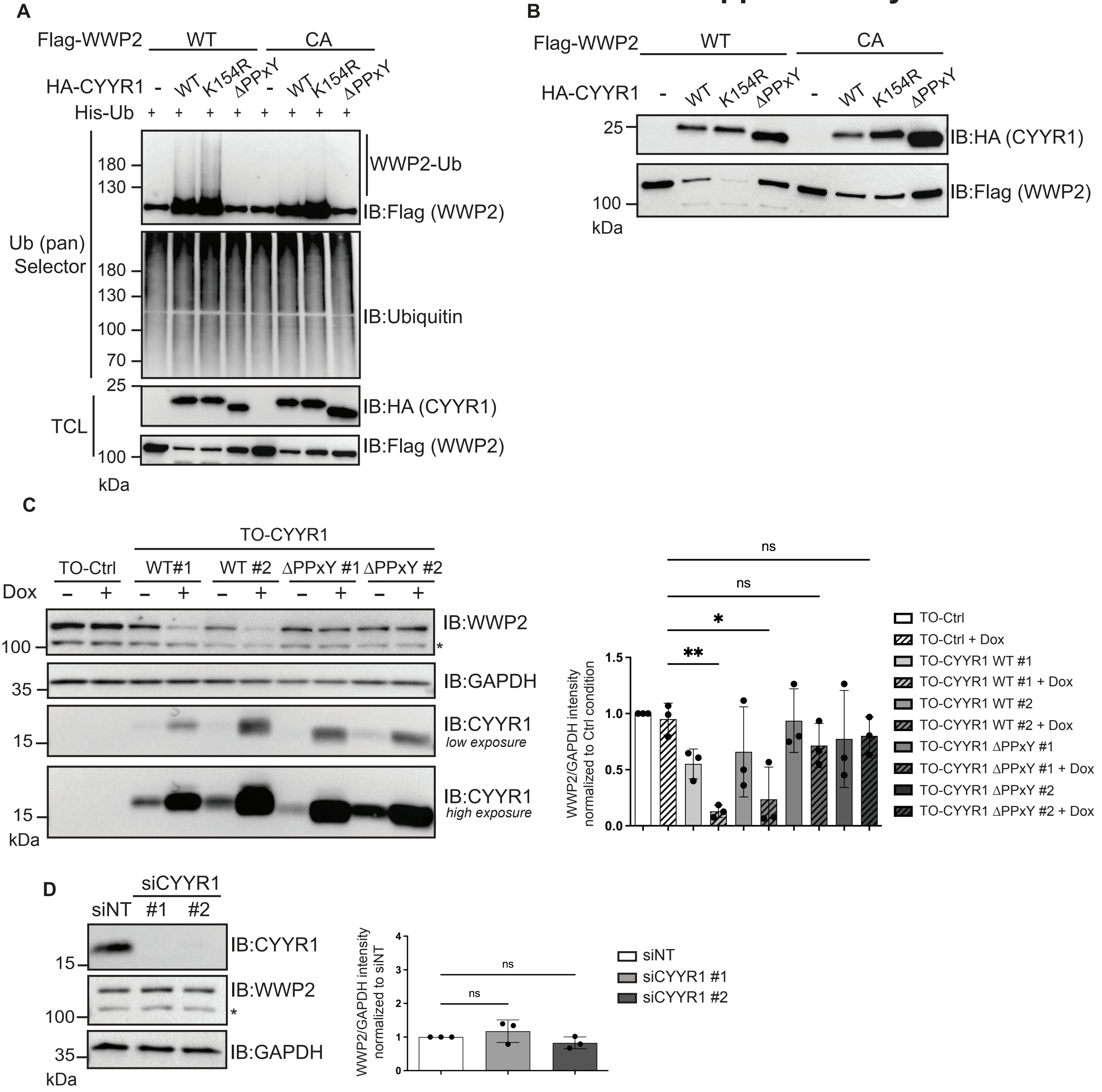

Figure S4: CYYR1 regulates WWP2 auto-ubiquitiation and protein level

(A) CYYR1 increases WWP2 auto-ubiquitination. HEK293 cells were transfected with His-Ub and Flag-WWP2-WT or Flag-WWP2-CA either alone or with different constructs for HA-CYYR1 as indicating. Cell lysates were pulled-down with Ubiquitin pan Selector resin and analyzed by western-blotting with the indicated antibodies. Western-blotting on total cell lysates (TCL) are shown as a transfection control. **(B)** CYYR1 decreases WWP2 protein level. Cell lysates form HEK293 cells transfected with Flag-WWP2-WT or Flag-WWP1-CA either alone or with different HA-CYYR1 constructs were analyzed by western-blotting with anti-Flag or anti-HA antibody. **(C)** CYYR1 decreases endogenous WWP2 protein level. MDA-MB-231 cells expressing doxycycline (Dox)-inducible CYYR1-WT or CYYR1-ΔPPxY (TO- CYYR1-WT and TO-CYYR1-ΔPPxY clones #1 and #2) were treated with 10 ng/mL of Dox during 24h. Cell lysates were analyzed by western-blotting with corresponding antibodies. Low and high exposure of the anti-CYYR1 western-blot is shown to highlight CYYR1 expression leakiness in absence of Dox. Statistical analysis was performed with one-way ANOVA followed by Dunnett's test(n=3). **(D)** CYYR1 depletion has no effect on WWP2 protein level in MDA-MB-468 cells. Lysates from MDA-MB-468 cells transfected with a non-targeting siRNA control (siNT) or two independent siRNA (#1 or #2) targeting CYYR1 were analyzed by western-blotting. Statistical analysis was performed with one-way ANOVA followed by Dunnett's test (n=3).

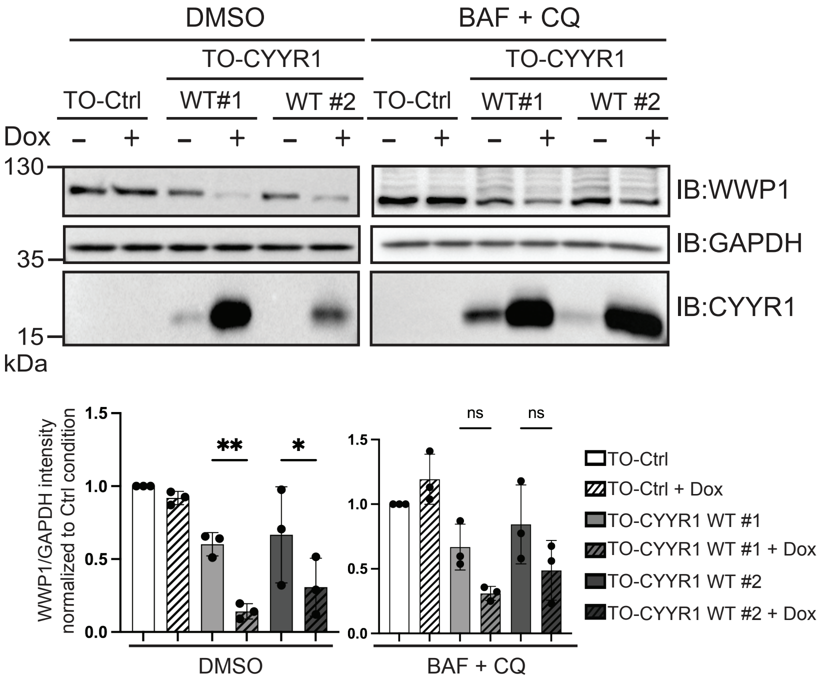

Figure S5: Lysosome inhibition attenuates WWP1 degradation induced by CYYR1
MDA-MB-231 TO-Ctrl or TO-CYYR1-WT clones #1 and #2 were treated with Dox and with Bafilomycin A1 (BAF) 100 nM and Chloroquine (CQ) 50 µM or DMSO for 24h before lysis and western-blotting with the indicated antibodies. Quantifications of the WWP1 intensity relative to GAPDH in each condition were normalized to the TO-Ctrl-Dox condition and p-values were calculated with one-way ANOVA followed by Sidak's test (n=3).

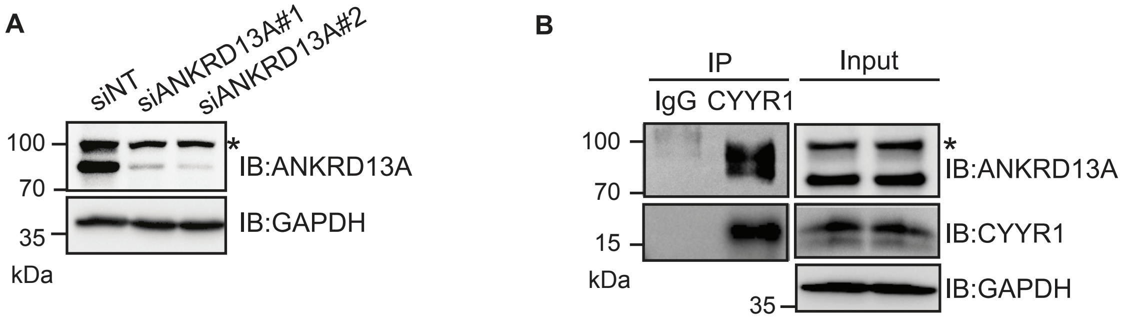

Figure S6: CYYR1 interacts with ANKRD13A

(A) ANKRD13A antibody validation. Protein lysates from MDA-MB-231 cells transfected with two independent siRNA targgeting ANKRD13A were analyzed by western-blotting with ANKRD13A antibody and GAPDH antibody as a loading control. **(B)** CYYR1 interacts with ANKRD13A. MDA-MB-468 cell lysate immunoprecipitated with anti-CYYR1 or IgG antibody were analyzed by western-blotting using the indicated antibodies. * indicates a non-specific band.

Table S1. List of the differentially enriched proteins in GFP-CYYR1 compared to GFP

13/5039 proteins	Gene & Synonyms	GFP-CYYR1/GFP								Total peptides in set	MW (kDa)	Description	Species
		Ratio	Log2 (Ratio)	Adj. p-value	CV %	Dist. pept. used	Pept. used	Pep. filter	Distrib. pep. in replic.				
O00308	**WWP2**	1000	1000			19	92	17	17, 17, 20, 19, 19/0, 0, 0, 0, 0	92	98,9	NEDD4-like E3 ubiquitin-protein ligase WWP2	Homo sapiens
P13646	KRT13	1000	1000			10	28	3	12, 3, 3, 4, 6/0, 0, 0, 0, 0	28	49,6	Keratin, type I cytoskeletal 13	Homo sapiens
P46379	BAG6,BAT3,G3	1000	1000			3	15	3	3, 3, 3, 3, 3/0, 0, 0, 0, 0	15	119,4	Large proline-rich protein BAG6	Homo sapiens
Q6KB66	KRT80,KB20	1000	1000			4	16	3	3, 3, 3, 3, 4/0, 0, 0, 0, 0	16	50,5	Keratin, type II cytoskeletal 80	Homo sapiens
Q6ZN17	LIN28B,CSDD2	2,44459	1,28959	0,02844	31,536	3	39	4	4, 4, 4, 4, 4/4, 3, 4, 4, 4	40	27,1	Protein lin-28 homolog B	Homo sapiens
Q6ZRV2	FAM83H	1000	1000			4	20	3	3, 3, 3, 3, 3/1, 1, 1, 1, 1	20	127,1	Protein FAM83H	Homo sapiens
Q8IZ07	**ANKRD13A,ANKRD13**	1000	1000			3	15	3	3, 3, 3, 3, 3/0, 0, 0, 0, 0	15	67,6	Ankyrin repeat domain-containing protein 13A	Homo sapiens
Q8TEP8	CEP192,KIAA1569,PP8407	1000	1000			3	15	3	3, 3, 3, 3, 3/0, 0, 0, 0, 0	15	279,1	Centrosomal protein of 192 kDa	Homo sapiens
Q96HA7	TONSL,IKBR,NFKBIL2	1000	1000			5	21	4	4, 5, 4, 4, 4/0, 0, 0, 0, 0	21	150,9	Tonsoku-like protein	Homo sapiens
Q96J02	**ITCH**	2,43865	1,28608	4,3E-35	4,8199	12	115	10	12, 13, 13, 10, 12/11, 10, 9, 13, 12	129	102,8	E3 ubiquitin-protein ligase Itchy homolog	Homo sapiens
Q96J86	**CYYR1,C21orf95**	1000	1000			4	35	7	7, 7, 7, 7, 7/0, 0, 0, 0, 0	35	16,6	Cysteine and tyrosine-rich protein 1	Homo sapiens
Q9H0M0	**WWP1**	1000	1000			23	124	23	23, 23, 25, 26, 27/0, 0, 0, 0, 0	124	105,2	NEDD4-like E3 ubiquitin-protein ligase WWP1	Homo sapiens
Q9UI43	MRM2,FJH1,FTSJ2	1000	1000			4	20	3	3, 3, 3, 3, 3/1, 1, 1, 1, 1	20	27,4	rRNA methyltransferase 2, mitochondrial	Homo sapiens

Proteins with a fold change ⩾ 2, p-value ⩽ 0.05 that display at least 3 peptides in each of the 5 replicate experiments have been selected.

Proteins that display no common peptides in the GFP condition (infinite ratio) are indicated with an arbitrary ratio value of 1000.

Table S2. Histopathological and clinical characteristics of 505 breast cancer patients

	Number of patients (%)	Number with metastases (%)	p-value [a]
Total	505 (100)	203 (40.2)	
Age			
≤50	119 (23.6)	52 (43.7)	0.49 (NS)
>50	386 (76.4)	151 (39.1)	
SBR histological grade [b, c]			
I	59 (12.0)	12 (20.3)	**0.0012**
II	230 (46.7)	97 (42.2)	
III	203 (41.3)	91 (44.8)	
Lymph node status [d]			
0	150 (29.9)	47 (31.3)	**<0.0001**
1-3	243 (48.5)	85 (35.0)	
>3	108 (21.6)	69 (63.9)	
Macroscopic tumor size [e]			
<25mm	239 (48.3)	74 (31.0)	**<0.0001**
>25mm	256 (51.7)	128 (50.0)	
ERα status			
Negative	171 (33.9)	73 (42.7)	0.14 (NS)
Positive	334 (66.1)	130 (38.9)	
PR status			
Negative	243 (48.1)	105 (43.2)	0.049
Positive	262 (51.9)	98 (37.4)	
ERBB2 status			
Negative	384 (76.0)	151 (39.3)	0.46 (NS)
Positive	121 (24.0)	52 (43.0)	
Molecular subtypes			
HR- ERBB2-	96 (19.0)	37 (38.5)	0.085 (NS)
HR- ERBB2+	68 (13.5)	34 (50.0)	
HR+ ERBB2-	288 (57.0)	114 (39.6)	
HR+ ERBB2+	53 (10.5)	18 (34.0)	
PIK3CA mutation status [f]			
wild type	341 (67.8)	142 (41.6)	0.18 (NS)
Mutated	162 (32.2)	60 (37.0)	

a Log-rank test (500 samples with MFS>6 months). NS: not significant
b Scarff Bloom Richardson
c Information available for 492 patients
d Information available for 501 patients
e Information available for 495 patients
f Information available for 503 patients
